# Colonic inflammation triggers **β** cell proliferation during obesity development via a liver-to-pancreas interorgan mechanism

**DOI:** 10.1172/jci.insight.183864

**Published:** 2025-05-08

**Authors:** Haremaru Kubo, Junta Imai, Tomohito Izumi, Masato Kohata, Yohei Kawana, Akira Endo, Hiroto Sugawara, Junro Seike, Takahiro Horiuchi, Hiroshi Komamura, Toshihiro Sato, Shinichiro Hosaka, Yoichiro Asai, Shinjiro Kodama, Kei Takahashi, Keizo Kaneko, Hideki Katagiri

**Affiliations:** Department of Diabetes, Metabolism and Endocrinology, Tohoku University Graduate School of Medicine, Sendai, Japan.

**Keywords:** Endocrinology, Metabolism, Beta cells, Obesity

## Abstract

Under insulin-resistant conditions, such as obesity, pancreatic β cells adaptively proliferate and secrete more insulin to prevent blood glucose elevation. We previously reported hepatic ERK activation during obesity development to stimulate a neuronal relay system, consisting of afferent splanchnic nerves from the liver and efferent vagal nerves to the pancreas, thereby triggering adaptive β cell proliferation. However, the mechanism linking obesity with the interorgan system originating in hepatic ERK activation remains unclear. Herein, we clarified that colonic inflammation promotes β cell proliferation through this interorgan system from the liver to the pancreas. First, dextran sodium sulfate (DSS) treatment induced colonic inflammation and hepatic ERK activation as well as β cell proliferation, all of which were suppressed by blockades of the neuronal relay system by several approaches. In addition, treatment with anti–lymphocyte Peyer’s patch adhesion molecule-1 (anti-LPAM1) antibody suppressed β cell proliferation induced by DSS treatment. Importantly, high-fat diet (HFD) feeding also elicited colonic inflammation, and its inhibition by anti-LPAM1 antibody administration suppressed hepatic ERK activation and β cell proliferation induced by HFD. Thus, colonic inflammation triggers adaptive β cell proliferation via the interorgan mechanism originating in hepatic ERK activation. The present study revealed a potentially novel role of the gastrointestinal tract in the maintenance of β cell regulation.

## Abstract 2

Colonic inflammation is both sufficient and necessary for the promotion of liver-to-pancreas neuronal relay-mediated adaptive β-cell proliferation during obesity, originating from hepatic ERK activation.

## Graphical abstract

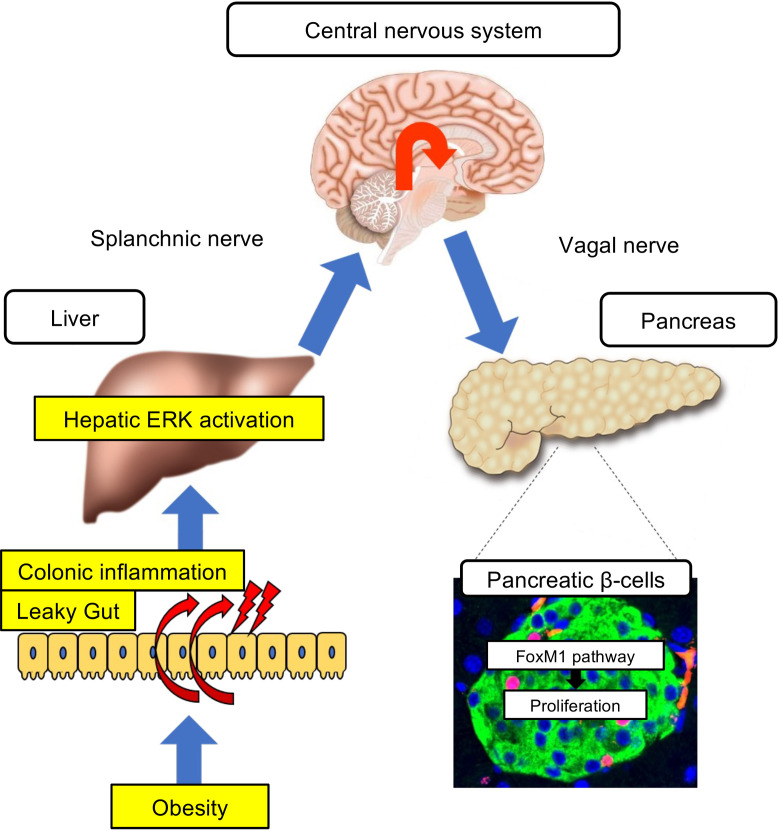

## Introduction

Under insulin-resistant conditions, such as obesity, pancreatic β cells are well known to adaptively proliferate and secrete more insulin to prevent blood glucose elevations (1). Therefore, these responses are regarded as important for maintaining glucose homeostasis and preventing diabetes development. It was previously reported that humoral factors, such as glucose (2), insulin (3) and Serpin B1 (4), can regulate β cell proliferation in such responses.

**Figure 1:**
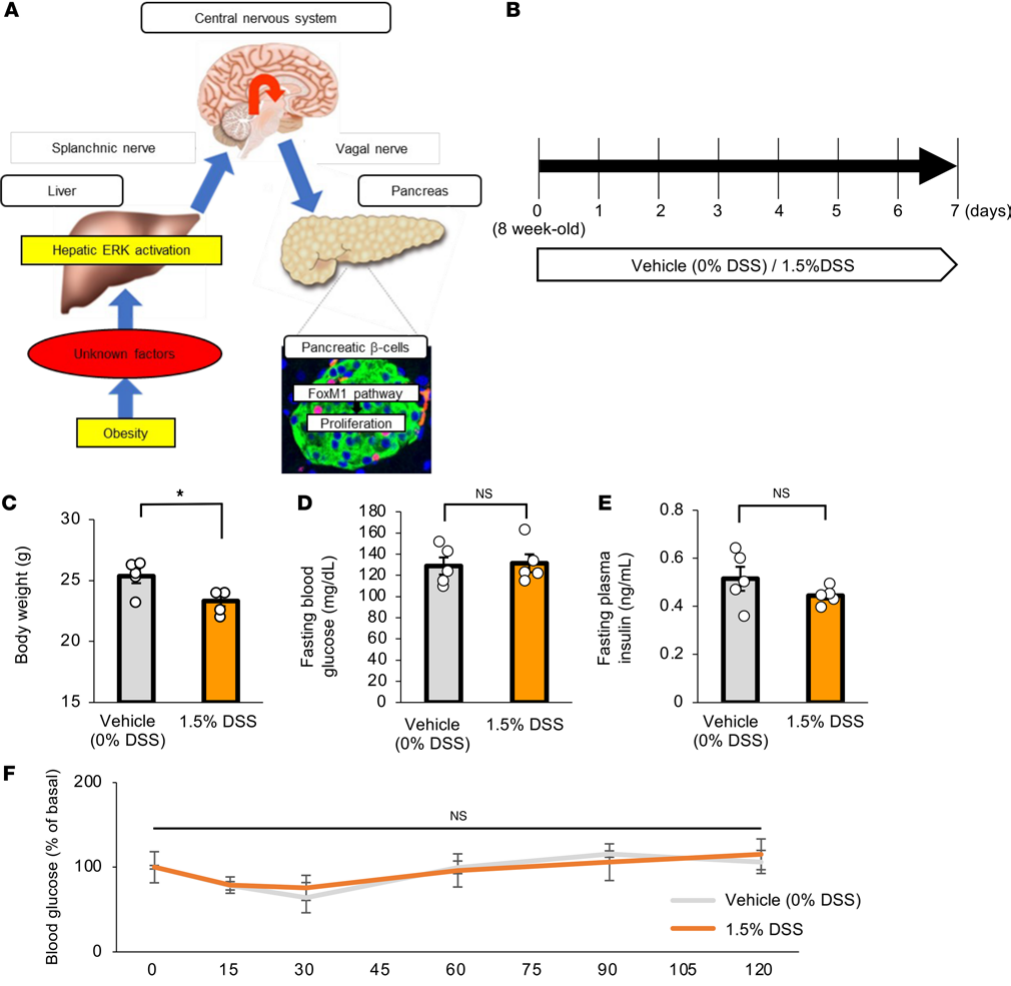
Metabolic phenotypes of mice treated with DSS. (**A**) Established schematic model of the neuronal relay system from the liver to the pancreas. (**B**) Scheme of the experimental plan for DSS administration. Mice were treated with 0% (vehicle) or 1.5% DSS (*n* = 5 per group). (**C**) Body weights at day 7 of experimental groups treated with 0% or 1.5% DSS (*n* = 5 per group). (**D**) Fasting blood glucose levels of mice at 7 days after vehicle or DSS treatment are shown (*n* = 5 per group). (**E**) Fasting insulin levels of mice at 7 days after vehicle or DSS treatment are shown (*n* = 5 per group). (**F**) The results of insulin tolerance tests after vehicle or DSS treatment are shown (*n* = 5 per group). Data are presented as means ± SEM. *P < 0.05 as assessed by the unpaired 2-tailed *t* test.

In addition to these mechanisms mediated by humoral factors, neuronal signals, especially those transmitted via the vagal nerves, are known to be regulators of both the functions (5–8) and the proliferation (5, 7–11) of β cells. In particular, hepatic activation of ERK was found to markedly induce β cell proliferation (5). Via this mechanism, liver-derived signals elicited by hepatic ERK activation are transmitted to the central nervous system (CNS) through afferent splanchnic nerves, then relayed to β cells through the efferent pancreatic vagal nerves (5) (Figure 1A). Notably, ERK signaling is activated in the livers of several murine models of obesity (12), and blockade of this interorgan system by expression of a dominant-negative mutant of mitogen-activated protein kinase/ERK kinase-1 (d/nMEK), pharmacological deafferentation of the splanchnic nerve, or dissection of the vagus was each shown to suppress the obesity-induced increases in β cells (5). Selective innervation of vagal nerve branches to islets in the pancreas was shown to be the anatomical basis of exclusive β cell proliferation (11). As for the underlying molecular mechanism, the Forkhead box M1 (FoxM1) pathway is involved in cellular proliferation (12). Furthermore, selective stimulation of the vagus innervating the pancreas alone enables β cells to proliferate in vivo (7). Thus, an interorgan system from the liver to the pancreas plays an important role in adaptive proliferation of β cells in settings of obesity (Figure 1A). However, the mechanism linking obesity with this neuronal system of hepatic ERK activation-induced β cell proliferation has yet to be clarified.

Recent evidence indicated that inflammation is provoked in portions of the gastrointestinal (GI) tract in settings of obesity. Notably, colonic inflammation is induced by alteration of the colonic environment, such as dysbiosis (13), leading to intestinal barrier disruption, which is widely referred to as “leaky gut” (14). The so-called leaky gut was recently reported to play important roles in the pathogenesis of several obesity-related metabolic disorders, such as fatty liver (15), by increasing levels of inflammatory factors, including lipopolysaccharide (LPS) and pro-inflammatory cytokines, in the portal vein (16). Interestingly, these pro-inflammatory factors are known to activate the ERK pathway in several cell types, including hepatocytes (17, 18). Therefore, we hypothesized that colonic inflammation plays an important role in hepatic activation of ERK, which serves as the trigger for adaptive β cell proliferation during obesity development.

Dextran sodium sulfate (DSS) is widely used for generating murine models of bowel inflammation and for eliciting the so-called leaky gut (19). Therefore, using DSS-treated mice as a model in which colonic inflammation is simply elicited without inducing obesity, we first attempted to determine whether intestinal barrier disruption itself evoked by colonic inflammation can trigger hepatic ERK activation and β cell proliferation via the interorgan system from the liver to the pancreas. We also examined the effects of blocking colonic inflammation on hepatic ERK activity and the effects of blocking each point of the interorgan system from the liver to the pancreas on β cell proliferation in DSS-treated mice. We then explored the involvement of colonic inflammation in hepatic ERK activation and β cell proliferation during obesity development using high-fat diet–treated (HFD-treated) mice. Using these strategies, we demonstrated that colonic inflammation promotes β cell proliferation through the neuronal relay system from the liver to the pancreas during obesity development.

## Results

### Colonic inflammation and colonic intestinal barrier disruption are induced in mice treated with DSS.

First, we examined the effects of DSS administration on systemic conditions in mice, including colonic inflammation. Mice were given free access to water containing a 1.5% concentration of DSS, as reported previously (19) (Figure 1B). Body weights of mice treated with DSS (DSS-mice) were significantly lower than those of vehicle-treated mice (vehicle-mice) after 7 days of being administered DSS (Figure 1C), while fasting blood glucose levels, fasting plasma insulin levels, and insulin sensitivity evaluated by insulin tolerance testing were similar in DSS- and vehicle-mice (Figure 1, D–F).

**Figure 2:**
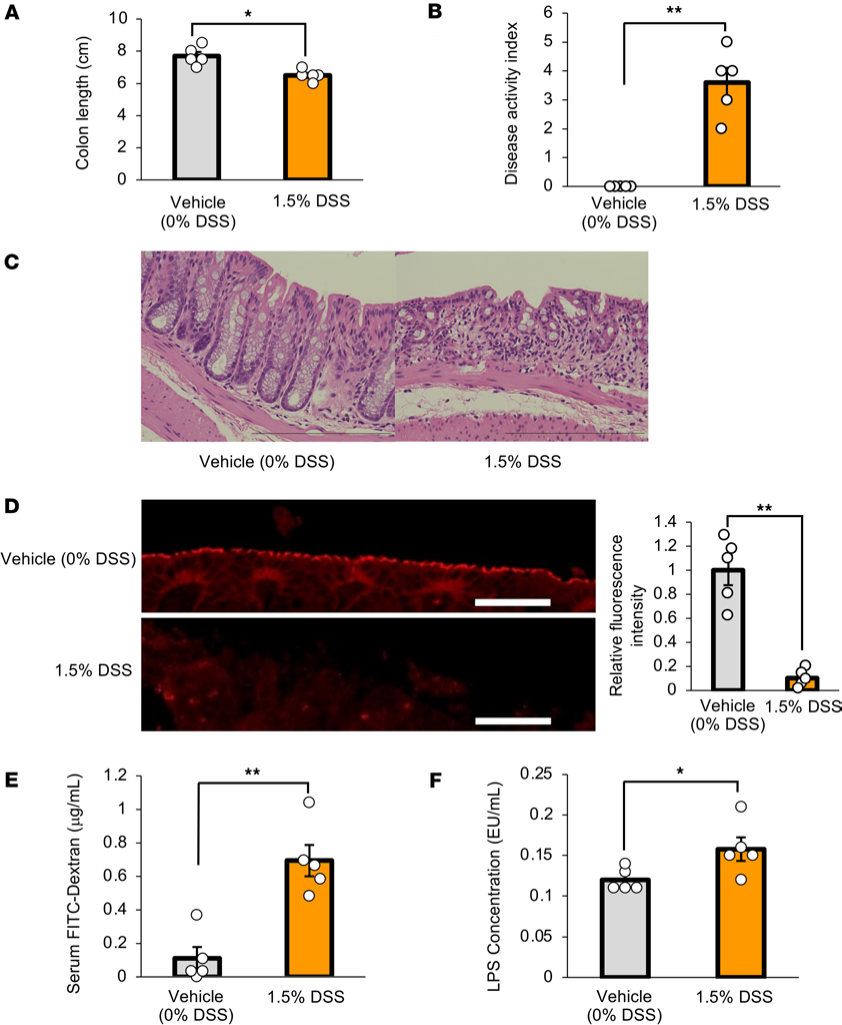
Colonic inflammation and colonic intestinal barrier disruption are induced in mice treated with DSS. (**A**) Colonic length is shown for each experimental group (*n* = 5 per group). (**B**) Disease activity indices are shown for each experimental group (*n* = 5 per group). (**C**) Photomicrograph image of hematoxylin and eosin staining on day 7 post-DSS. Epithelial and crypt alignment of the distal colon were apparently impaired. Scale bars indicate 100 μm. (**D**) Photomicrograph image of the occludin immunofluorescence in the distal colon, and the relative fluorescence intensity in each experimental group was calculated (*n* = 5 per group). Scale bars indicate 100 μm. (**E**) Serum FITC-Dextran levels (40 kDa) after administration to mice in each of the experimental groups (*n* = 5 per group). (**F**) Serum LPS concentrations in the portal veins of mice in each of the experimental groups (*n* = 5 per group). Data are presented as means ± SEM. *P < 0.05, **P < 0.01 as assessed by the unpaired 2-tailed *t* test.

DSS administration reportedly promotes colonic inflammation along with shortening the colon (19, 20). As such, DSS-mice had significantly shorter colons than vehicle-mice after 7 days of DSS administration (Figure 2A). The disease activity index, which is determined based on body weight, diarrhea, and bloody stools, is widely used for assessing the severity of colonic inflammation (19). As expected, the disease activity index was significantly increased in DSS-mice (Figure 2B). These results verified colonic inflammation had been induced in DSS-mice. Next, we histologically evaluated colonic inflammation after 7 days of DSS administration. In DSS-mice, disruption of epithelial layers and ductal structures as well as infiltration of mononuclear cells were observed mainly in the distal colon (Figure 2C). Then, we examined whether intestinal barrier disruption was elicited in the colons of DSS-mice. Expression of occludin, a tight junction–associated protein used as a marker of intestinal barrier dysfunction, in colonic epithelium was significantly decreased in the distal colon in DSS-mice (Figure 2D). Furthermore, the concentration of orally administered FITC-Dextran in the portal vein, which is widely used for evaluating intestinal permeability (21), was significantly higher in DSS-mice than in vehicle-mice (Figure 2E). Moreover, the concentration of LPS in the portal vein, which is reportedly increased as a result of leaky gut (22), was also elevated in DSS-mice (Figure 2F). Collectively, these observations showed increased intestinal permeability, in parallel with colonic inflammation, to have been induced in DSS-mice.

### Hepatic ERK pathway activation and β cell proliferation are enhanced in mice treated with DSS.

**Figure 3:**
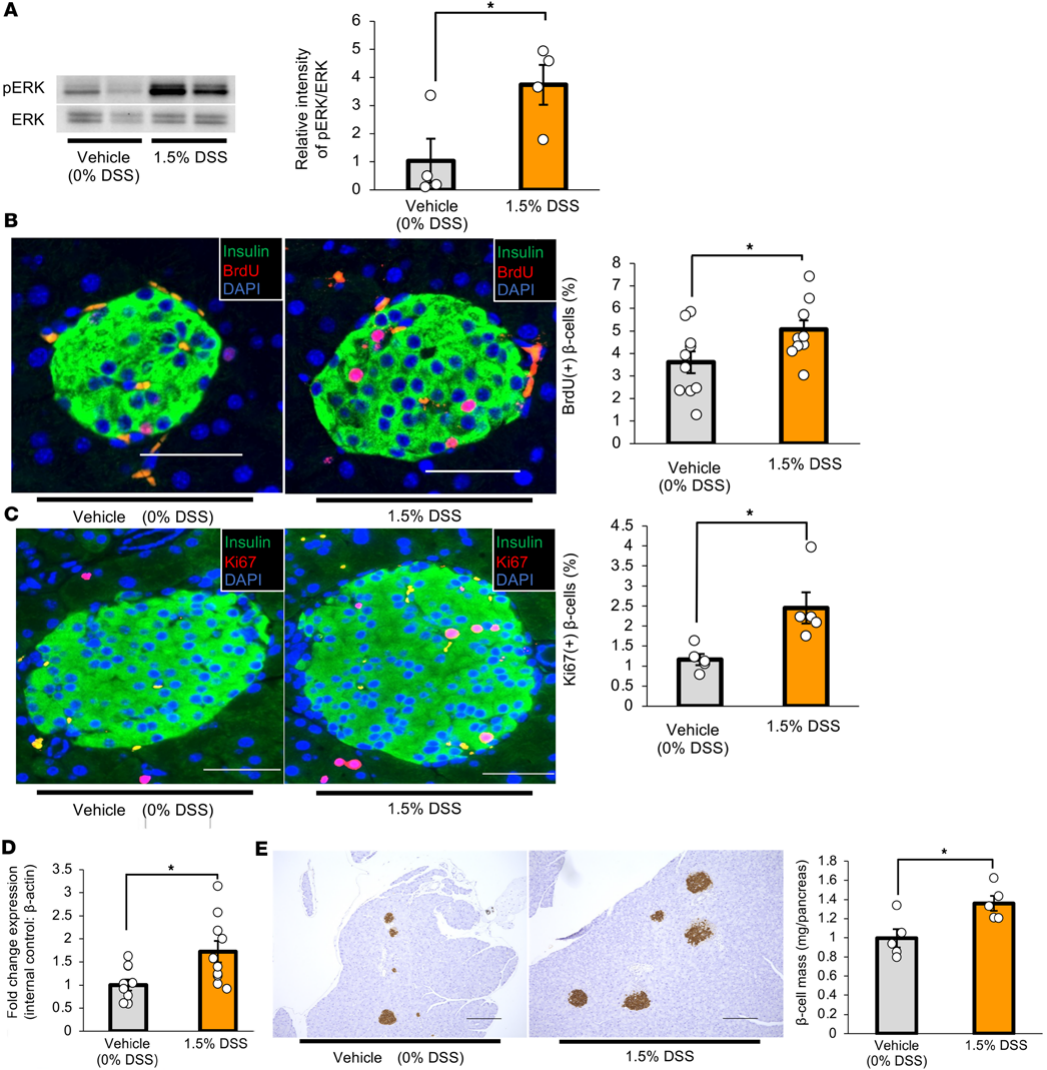
Hepatic ERK pathway activation and β cell proliferation are enhanced in mice treated with DSS. (**A**) Representative images and calculated relative intensities of liver extract immunoblotting with anti-ERK and phosphorylated ERK (pERK) of vehicle- or DSS-treated mice on day 7 (*n* = 4 per group). Band intensities were calculated as pERK intensity per ERK intensity. (**B**) Representative images and counted BrdU-positive β cell percentage in each group are shown (*n* = 10 per group). Scale bars indicate 50 μm. (**C**) Representative images and counted Ki67-positive β cell percentage at 5 days after vehicle or DSS administration (*n* = 5 per group). Scale bars indicate 50 μm. (**D**) Expression levels of *FoxM1* gene in islets isolated from mice 5 days after vehicle or DSS administration (*n* = 10 per group). (**E**) Representative images and measured β cell masses after 7 days of vehicle or DSS treatment (*n* = 5 per group). Scale bars indicate 200 μm. Data are presented as means ± SEM. *P < 0.05 as assessed by the unpaired 2-tailed *t* test.

Next, we examined whether the hepatic ERK pathway is activated in DSS-mice. Western blotting revealed that phosphorylated ERK protein was significantly increased in the livers of DSS-mice, as compared with vehicle-mice, after 7 days of DSS administration (Figure 3A). In addition, we histologically examined β cell proliferation employing BrdU incorporation during the 7-day DSS administration period and found that BrdU-positive β cells were significantly increased in DSS-mice (Figure 3B). We further examined β cell proliferation by another procedure, Ki67 staining, and obtained consistent results (Figure 3C). These findings suggest that colonic inflammation promotes both hepatic ERK activation and β cell proliferation. FoxM1 is a critical transcription factor in cell cycle progression (23) and plays important roles in adaptive β cell proliferation during obesity mediated by the interorgan system linking the liver to the pancreas (7, 11). Expression of *FoxM1* was significantly increased in the islets isolated from DSS-mice after 5 days of DSS administration, as compared with those from vehicle-mice (Figure 3D). As a result, β cell mass was significantly increased in DSS-mice after 7 days of DSS administration (Figure 3E). These findings suggest DSS treatment to promote β cell proliferation through a mechanism involving β cell FoxM1 signaling.

### Suppression of the hepatic ERK pathway blocks β cell proliferation in DSS-mice.

**Figure 4:**
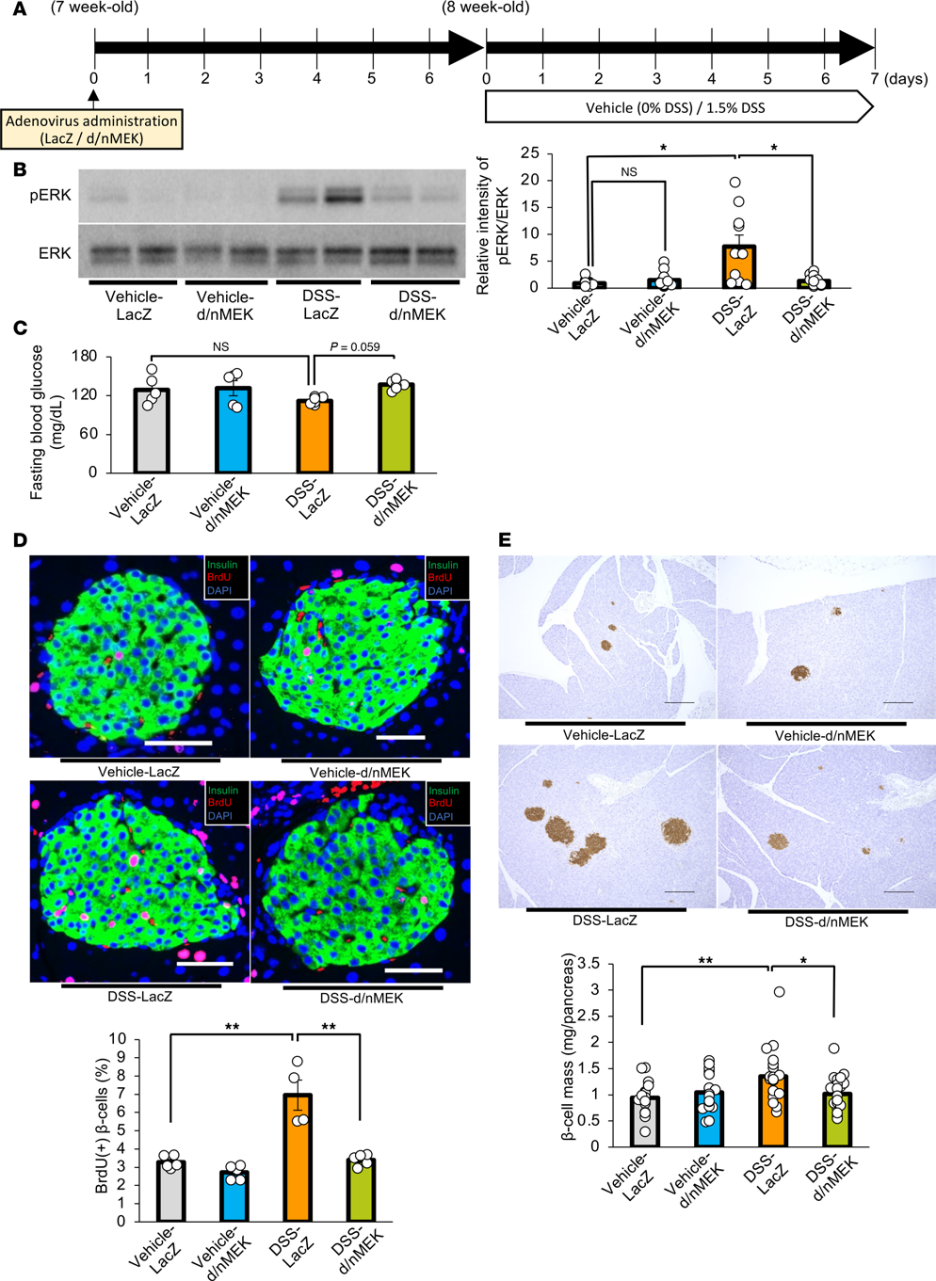
Suppression of the hepatic ERK pathway blunts β cell proliferation in mice treated with DSS. (**A**) Scheme of the experimental plan for DSS administration and adenovirus administration using LacZ or dominant-negative MEK (d/nMEK). (**B**) Representative images and calculated relative intensities of liver extract immunoblotting with anti-ERK and pERK on day 7 of each group (*n* = 10/12/10/12 per group). Band intensities were calculated as pERK intensity per ERK intensity. (**C**) Fasting blood glucose levels of mice in each group (*n* = 5/5/6/7 per group). (**D**) Representative images and counted BrdU-positive β cell percentage in each group (*n* = 5/5/4/5 per group). Scale bars indicate 50 μm. (**E**) Representative images and measured β cell masses are shown for each group (*n* = 15/18/20/23 per group). Scale bars indicate 200 μm. Data are presented as means ± SEM. *P < 0.05, **P < 0.01 as assessed by 1-way ANOVA, followed by Tukey’s honestly significant differences (HSD) post hoc test.

DSS administration may induce inflammatory responses in a variety of tissues besides activating the ERK pathway in the liver. Therefore, to examine whether hepatic ERK activation per se is involved in β cell mass augmentation, we expressed d/nMEK in the livers of DSS-mice employing the adenoviral gene transduction system (5), by injecting the adenoviruses 1 week prior to staring the DSS administration (Figure 4A). Although DSS administration reduced the body weights of our experimental animals, these weights did not differ between DSS-mice with d/nMEK and those given control LacZ adenoviruses after 7 days of DSS administration (Supplemental Figure 1A; supplemental material available online with this article; https://doi.org/10.1172/jci.insight.183864DS1). DSS-induced colonic shortening (Supplemental Figure 1B) as well as increases in the disease activity indices (Supplemental Figure 1C) were similar in d/nMEK-treated and LacZ adenovirus–treated mice. In addition, concentrations of orally administered FITC-Dextran in the portal vein were similar in DSS-mice given d/nMEK and control LacZ adenovirus (Supplemental Figure 1D). Thus, administration of d/nMEK adenovirus minimally affected colonic inflammation status.

As expected, enhancement of hepatic ERK phosphorylation after 7 days of DSS administration was significantly suppressed by administration of d/nMEK adenovirus, as compared with LacZ adenovirus, treatment (Figure 4B). The d/nMEK adenovirus–treated DSS-mice tended to exhibit higher fasting glucose levels than LacZ adenovirus–treated DSS-mice (Figure 4C). Strikingly, the increases in BrdU-positive β cells and β cell mass observed in control mice on day 7 after starting DSS administration were almost completely blocked in mice treated with d/nMEK adenovirus (Figure 4, D and E). Considering that colonic inflammation was similarly elicited in d/nMEK adenovirus– and LacZ adenovirus–treated DSS-mice, activation of the hepatic ERK pathway, rather than inflammation in other tissues, plays a major role in β cell proliferation triggered by DSS-induced colonic inflammation.

### Blockade of the neuronal relay system blunts β cell proliferation in DSS-mice.

**Figure 5:**
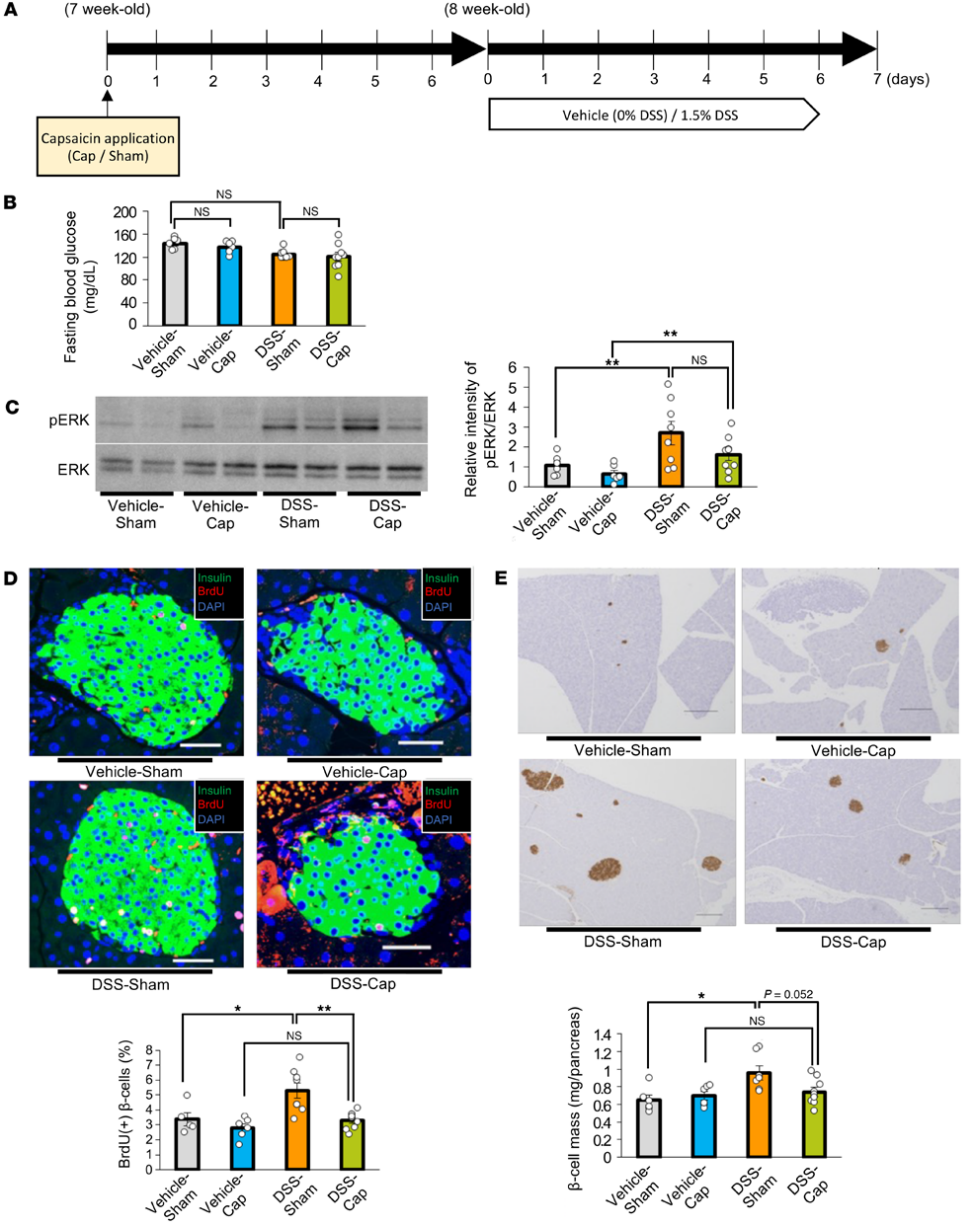
Blockade of the neuronal relay by pharmacologically blocking the afferent splanchnic nerve fibers blunts β cell proliferation in mice treated with DSS. (**A**) Scheme of the experimental plan for DSS administration and capsaicin treatment. (**B**) Fasting blood glucose levels of mice in each group (*n* = 6/6/8/9 per group). (**C**) Representative images and calculated relative intensities of liver extract immunoblotting with anti-ERK and pERK on day 7 for each group: Vehicle-Sham, Vehicle-Cap, DSS-Sham, and DSS-Cap (*n* = 6/6/8/9 per group). Band intensities were calculated as pERK intensity per ERK intensity. (**D**) Represedntative images and counted BrdU-positive β cell percentage in each group (*n* = 6/6/7/9 per group). Scale bars indicate 50 μm. (**E**) Representative images and measured β cell masses for each group (*n* = 6/6/7/9 per group). Scale bars indicate 200 μm. Data are presented as means ± SEM. *P < 0.05, **P < 0.01 as assessed by 1-way ANOVA, followed by Tukey’s HSD post hoc test.

Next, we investigated whether the liver-to-pancreas interorgan communication transmitted by the nervous system (Figure 1A) is indeed involved in β cell mass augmentation in DSS-mice by inhibiting afferent splanchnic and efferent vagal nerves. First, we administered DSS to mice that had undergone pharmacological blockade of the afferent splanchnic nerve fibers from the liver by application of capsaicin to this nerve (DSS-Cap) (5). Mice that had received a sham operation were used as controls (DSS-Sham). One week after the surgery, DSS administration was started, then continued for another week (Figure 5A). DSS administration similarly reduced body weights in these 2 groups of mice (Supplemental Figure 2A). Fasting blood glucose levels were similar in DSS-Sham and DSS-Cap (Figure 5B). On the other hand, DSS administration induced significant colonic shortening in both groups (Supplemental Figure 2B). Increases in the disease activity indices were similar in DSS-Sham and DSS-Cap (Supplemental Figure 2C). Additionally, capsaicin treatment did not affect DSS-induced increases in concentrations of orally administered FITC-Dextran in the portal vein (Supplemental Figure 2D). Collectively, these results suggest that capsaicin treatment minimally affects colonic inflammation and disruption of the intestinal barrier in DSS-mice.

Under such capsaicin-treated conditions, hepatic ERK phosphorylation after 7 days of DSS administration was similarly enhanced in DSS-Sham- and DSS-Cap-mice (Figure 5C). However, increases in BrdU-positive β cells after 7 days of DSS administration were almost completely blocked in DSS-Cap-mice (Figure 5D). In addition, increases in DSS-induced β cell mass were blocked in capsaicin-treated mice (Figure 5E). Thus, afferent splanchnic signals from the liver were verified to mainly contribute to β cell proliferation promoted by DSS administration.

**Figure 6:**
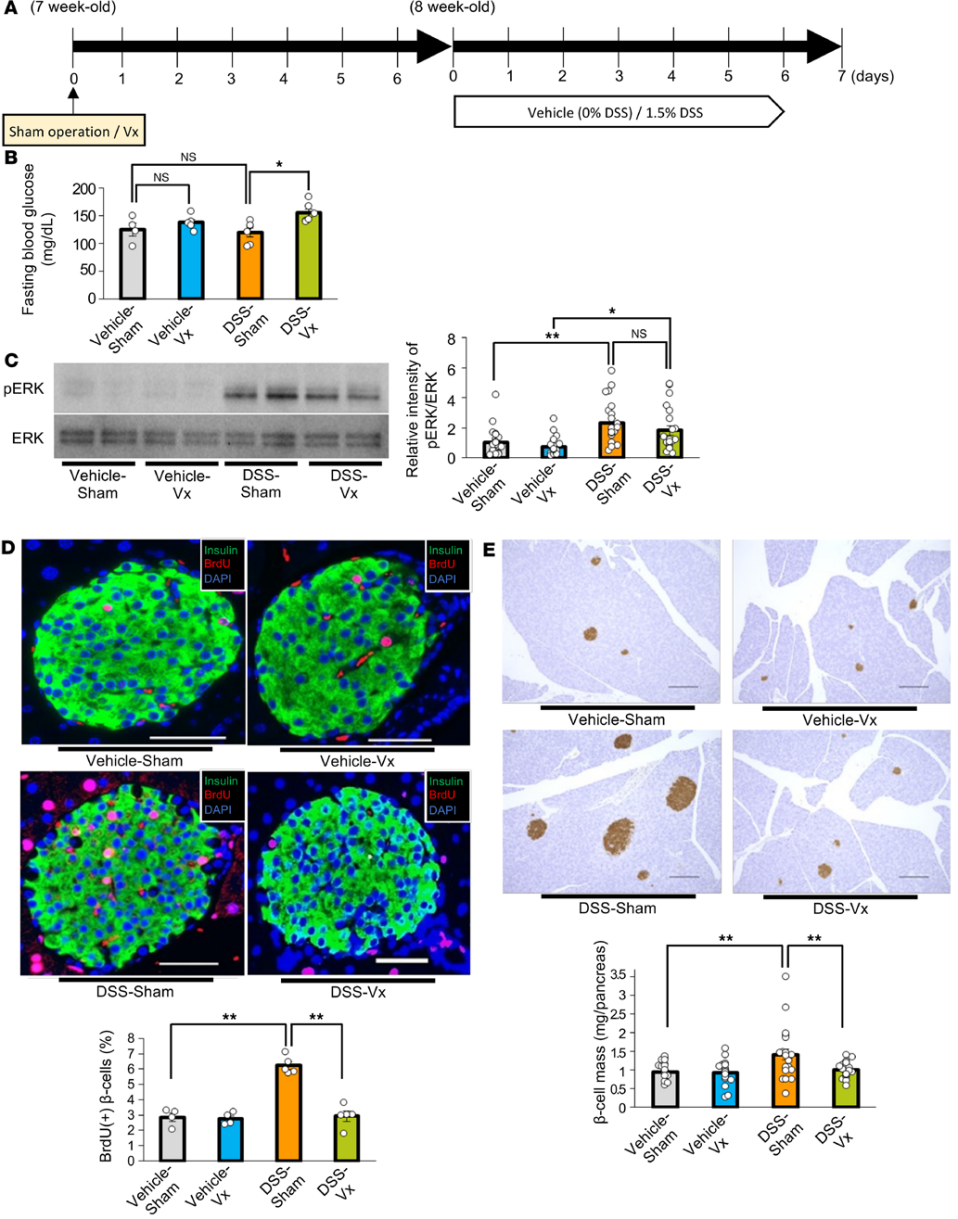
Blockade of the neuronal relay by subdiaphragmatic vagotomy blunts β cell proliferation in mice treated with DSS. (**A**) Scheme of the experimental plan for DSS administration and vagotomy (Vx). (**B**) Fasting blood glucose levels of mice in each group (*n* = 4/5/6/6 per group). (**C**) Representative images and calculated relative intensities of liver extract immunoblottings with anti-ERK and pERK for each group: Vehicle-Sham, Vehicle-Vx, DSS-Sham, and DSS-Vx (*n* = 23/19/23/21 per group). Band intensities were calculated as pERK intensity per ERK intensity. (**D**) Representative images and counted BrdU-positive β cell percentage in each group (*n* = 4/4/5/5 per group). Scale bars indicate 50 μm. (**E**) Representative images and measured β cell masses for each group (*n* = 23/20/23/21 per group). Scale bars indicate 200 μm. Data are presented as means ± SEM. *P < 0.05, **P < 0.01 as assessed by 1-way ANOVA, followed by Tukey’s HSD post hoc test.

Next, we administered DSS to mice that had undergone subdiaphragmatic vagotomy (DSS-Vx) (5, 9–11). Mice that had received a sham operation were used as controls (DSS-Sham). One week after the surgery, DSS administration was initiated, then continued for another week (Figure 6A). DSS administration similarly reduced body weights in these 2 groups of mice (Supplemental Figure 3A). Fasting blood glucose was significantly higher in DSS-Vx than in DSS-Sham (Figure 6B). While significant colonic shortening was observed in both groups of mice, slight but significant shortening further occurred in DSS-Vx as compared with DSS-Sham (Supplemental Figure 3B). These findings may reflect the suppressive effect of vagal nerves innervating the intestine on colonic inflammation, as reported previously (24). However, increases in the disease activity indices were similar in DSS-Sham- and DSS-Vx-mice (Supplemental Figure 3C). In addition, subdiaphragmatic vagotomy did not affect DSS-induced increases in concentrations of orally administered FITC-Dextran in the portal vein (Supplemental Figure 3D). These results indicate that, at a minimum, subdiaphragmatic vagotomy suppressed neither colonic inflammation nor disruption of the intestinal barrier in DSS-mice.

Under these experimental conditions, hepatic ERK phosphorylation after DSS administration was similarly enhanced in DSS-Sham- and DSS-Vx-mice (Figure 6C). Importantly, increases in BrdU-positive β cells after DSS administration were almost completely blocked in DSS-Vx-mice (Figure 6D). Consistently, increases in β cell mass after DSS administration were also blocked by subdiaphragmatic vagotomy (Figure 6E). Thus, vagal signals are involved in β cell proliferation promoted by DSS treatment. Taken together, colonic inflammation induces β cell proliferation mediated mainly by the liver-to-pancreas interorgan neuronal mechanism that reportedly leads to adaptive β cell proliferation during obesity development.

### Blockade of colonic inflammation suppresses hepatic ERK activation and β cell proliferation in DSS-mice.

We next attempted to block colonic inflammation employing anti–lymphocyte Peyer’s patch adhesion molecule-1 (LPAM1) antibody and analyzed the effect of colonic inflammation blockade on hepatocyte and β cell phenotypes. LPAM1, also known as integrin α_4_β_7_, is expressed on the T cell surface (25) and binds to mucosal addressin cell adhesion molecule-1 (MAdCAM1) specifically expressed on vascular endothelial cells of the colon, which in turn promotes colonic inflammation (26). Therefore, anti-LPAM1 antibody selectively inhibits LPAM1 binding to MAdCAM1 and specifically suppresses colonic inflammation in DSS-mice (27).

**Figure 7:**
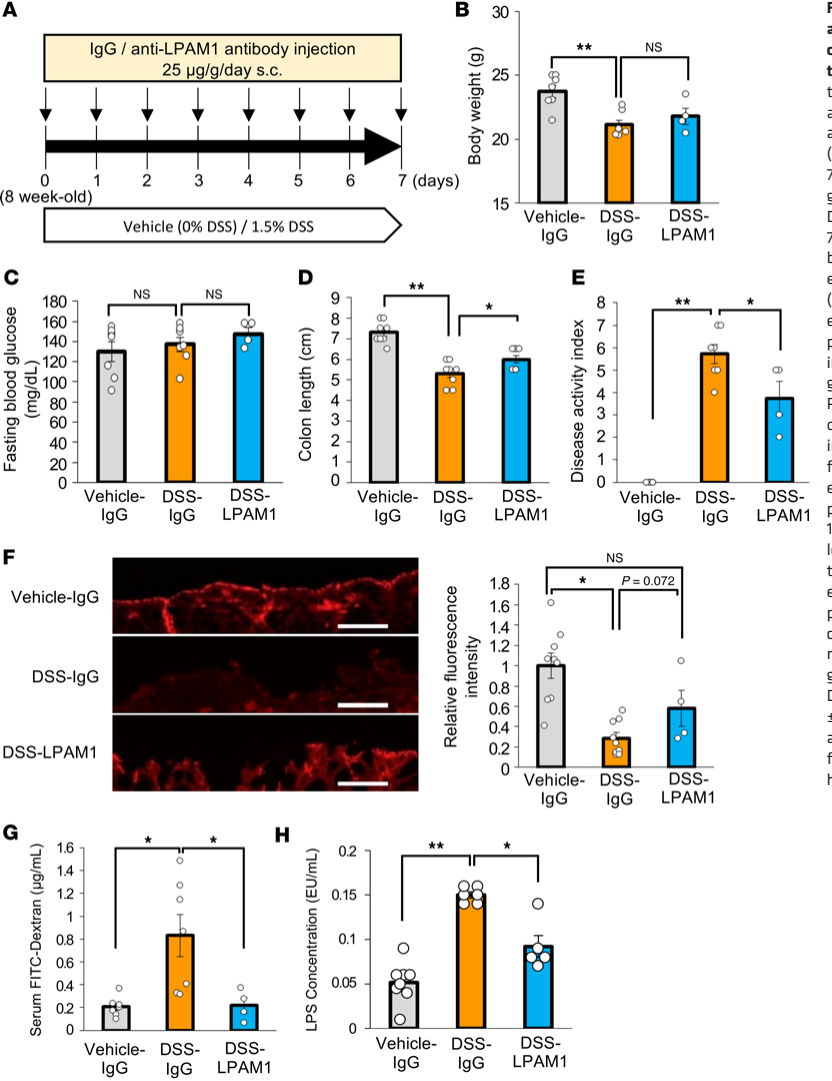
Administration of anti-LPAM1 antibody suppresses colonic inflammation in mice treated with DSS. (**A**) Scheme of the experimental plan for DSS administration and antibody administration subcutaneously (s.c.). (**B**) Body weights at day 7 for each of the experimental groups treated with Vehicle-IgG, DSS-IgG, or DSS-LPAM1 (*n* = 7/7/4 per group). (**C**) Fasting blood glucose levels of mice in each group (*n* = 7/7/4 per group). (**D**) Colonic lengths of each of the experimental groups (*n* = 8/8/8 per group). (**E**) Disease activity indices for each experimental group (*n* = 7/7/4 per group). (**F**) Photomicrograph image of the occludin immunofluorescence in the distal colon and relative fluorescence intensity in each experimental group (*n* = 9/9/4 per group). Scale bars indicate 100 μm. (**G**) Serum FITC-Dextran levels (40 kDa) after administration to mice in each of the experimental groups (*n* = 7/7/4 per group). (**H**) Serum LPS concentrations in the portal veins of mice in each of the experimental groups (*n* = 8/6/5 per group). Data are presented as means ± SEM. *P < 0.05, **P < 0.01 as assessed by 1-way ANOVA, followed by Tukey’s HSD post hoc test.

First, we examined the effects of anti-LPAM1 antibody on colonic inflammation. Mice given DSS were concomitantly treated with either control IgG or anti-LPAM1 antibody daily for 7 days (Figure 7A). DSS administration decreased body weights of these mice, and their weights were similar to those of DSS-mice treated with either anti-LPAM1 antibody (DSS-LPAM1) or control IgG (DSS-IgG) after 7 days of DSS administration (Figure 7B). Fasting blood glucose levels were also similar in DSS-LPAM1- and DSS-IgG-mice (Figure 7C). Meanwhile, colonic shortening induced by DSS was slightly but significantly reversed by administration of anti-LPAM1 antibody (Figure 7D). In addition, the disease activity index increase induced by DSS administration was significantly blunted by anti-LPAM1 antibody treatment (Figure 7E). These results indicate that anti-LPAM1 antibody treatment ameliorated colonic inflammation. Moreover, decreases in colonic occludin expression, as well as increases in orally administered FITC-Dextran and LPS concentrations in the portal vein induced by DSS, were significantly blunted by treatment with anti-LPAM1 antibody (Figure 7, F–H). These observations indicate that the intestinal barrier disruption in the colon was largely prevented by treatment with anti-LPAM1 antibody.

**Figure 8:**
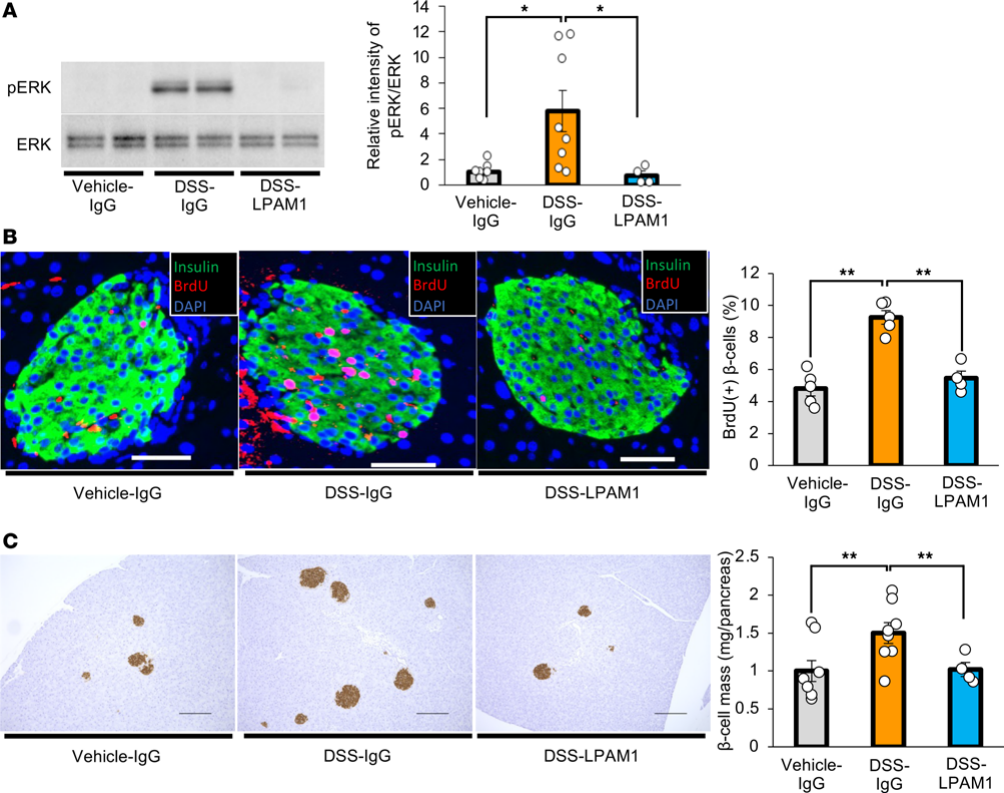
Blockade of colonic inflammation suppresses hepatic ERK activation and β cell proliferation in mice treated with DSS. (**A**) Representative images and calculated relative intensities of liver extract immunoblotting with anti-ERK and pERK on day 7 for each group. Band intensities were calculated as pERK intensity per ERK intensity (*n* = 8/8/4 per group). (**B**) Representative images and counted BrdU-positive β cell percentage in each group (*n* = 5/5/4 per group). Scale bars indicate 50 μm. (**C**) Representative images and measured β cell masses for each group (*n* = 8/8/4 per group). Scale bars indicate 200 μm. Data are presented as means ± SEM. *P < 0.05, **P < 0.01 as assessed by 1-way ANOVA, followed by Tukey’s HSD post hoc test.

Importantly, anti-LPAM1 antibody treatment significantly blunted hepatic ERK activation (Figure 8A) after 7 days of DSS administration. Furthermore, increases in BrdU-positive β cells and β cell mass after a 7-day DSS administration period were blocked by anti-LPAM1 antibody treatment (Figure 8, B and C). Collectively, these results indicate that suppression of inflammation specifically in the colon and the resulting preservation of its intestinal barrier blunted hepatic ERK activation and β cell mass expansion in DSS-mice. Thus, colonic inflammation per se may lead to pancreatic β cell proliferation with hepatic ERK activation by inducing disruption of the intestinal barrier.

### Colonic inflammation, hepatic ERK activation, and β cell proliferation are induced in mice with HFD-induced obesity.

**Figure 9:**
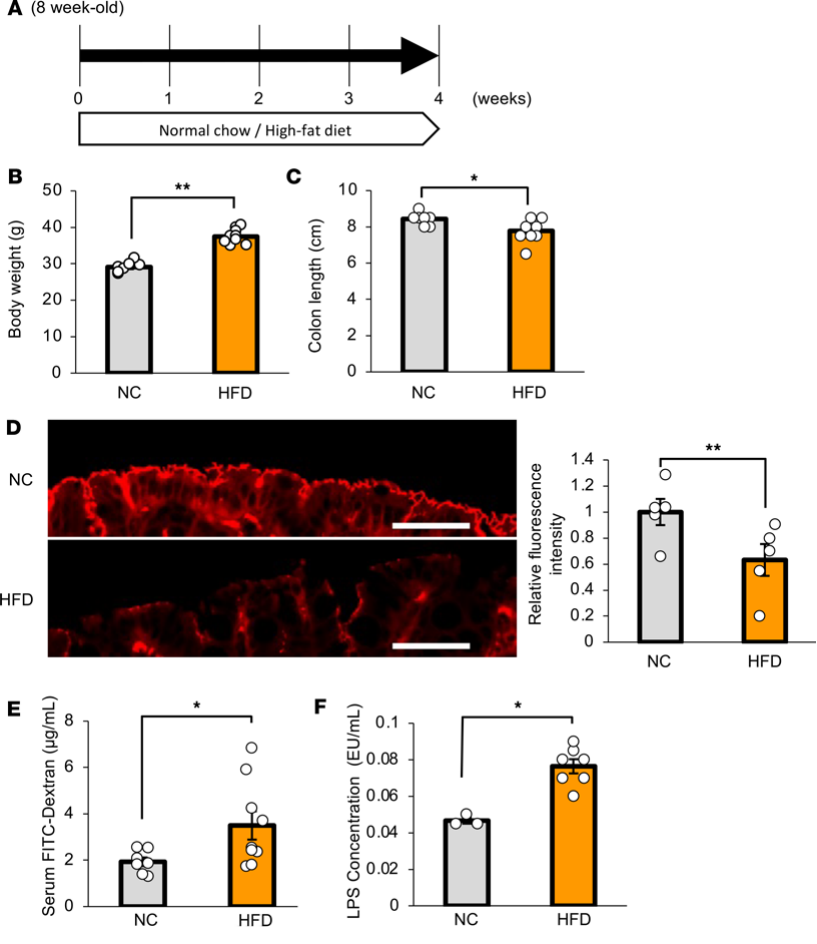
Colonic inflammation was induced in mice with HFD-induced obesity. (**A**) Scheme of the experimental plan for NC or HFD feeding. (**B**) Body weight at 4 weeks after feeding of NC or HFD in 8-week-old mice (*n* = 7/9 per group in NC and HFD mice). (**C**) Colonic lengths in each of the experimental groups (*n* = 7/9 per group). (**D**) Photomicrograph image of the occludin immunofluorescence in the distal colon and the relative fluorescence intensity in each experimental group (*n* = 5/5 per group). Scale bars indicate 100 μm. (**E**) Serum FITC-Dextran levels (4 kDa) after administration to mice in each experimental group (*n* = 7/9 per group). (**F**) Serum LPS concentrations in the portal veins of mice in each of the experimental groups (*n* = 3/7 per group). Data are presented as means ± SEM. *P < 0.05, **P < 0.01 as assessed by the unpaired 2-tailed *t* test.

**Figure 10:**
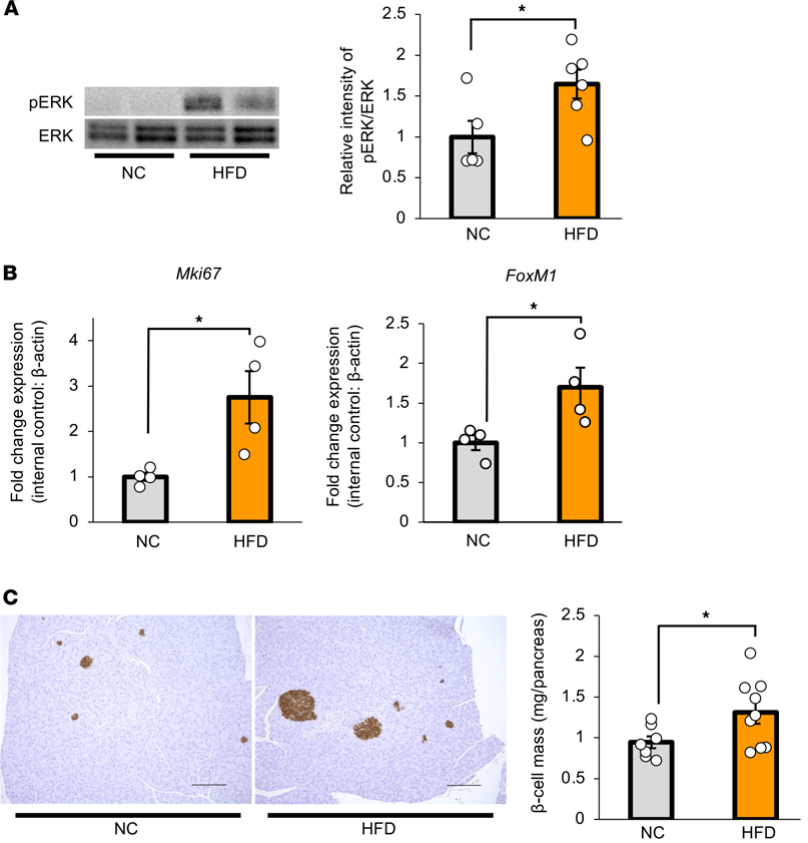
Hepatic ERK activation and β cell proliferation are induced in mice with HFD-induced obesity. (**A**) Representative images and calculated relative intensities of liver extract immunoblotting with anti-ERK and pERK in each group (*n* = 5/6 per group). Band intensities were calculated as pERK intensity per ERK intensity. (**B**) Expression levels of *FoxM1* and *Mki67* genes in islets isolated from mice in each group (*n* = 4/4 per group). (**C**) Representative images and measured β cell masses for each group (*n* = 7/9 per group). Scale bars indicate 200 μm. Data are presented as means ± SEM. *P < 0.05 as assessed by the unpaired 2-tailed *t* test.

Next, as a main focus of the present study, we investigated whether intestinal barrier disruption during obesity development is involved in adaptive β cell proliferation. We first examined colonic inflammation in mice with HFD-induced obesity that had been given an HFD for 4 weeks (HFD-mice), in comparison with that in normal chow–fed mice (NC-mice) (Figure 9A). Body weights of HFD-mice were significantly greater than those of NC-mice (Figure 9B). In contrast, the colonic lengths of HFD-mice were significantly shorter than those in NC-mice (Figure 9C), and colonic expression of occludin was significantly lower in HFD-mice (Figure 9D). Consistently, concentrations of orally administered FITC-Dextran and LPS in the portal vein were significantly higher in HFD-mice (Figure 9, E and F). Under these conditions, enhancement of hepatic ERK phosphorylation (Figure 10A), upregulation of *Mki67* and *FoxM1* gene expressions in isolated pancreatic islets (Figure 10B), and increased β cell mass (Figure 10C) were observed. Thus, HFD feeding induced β cell proliferation along with hepatic ERK activation, in accordance with colonic intestinal barrier disruption.

### Blockade of colonic inflammation suppresses hepatic ERK activation and β cell proliferation in mice with HFD-induced obesity.

**Figure 11:**
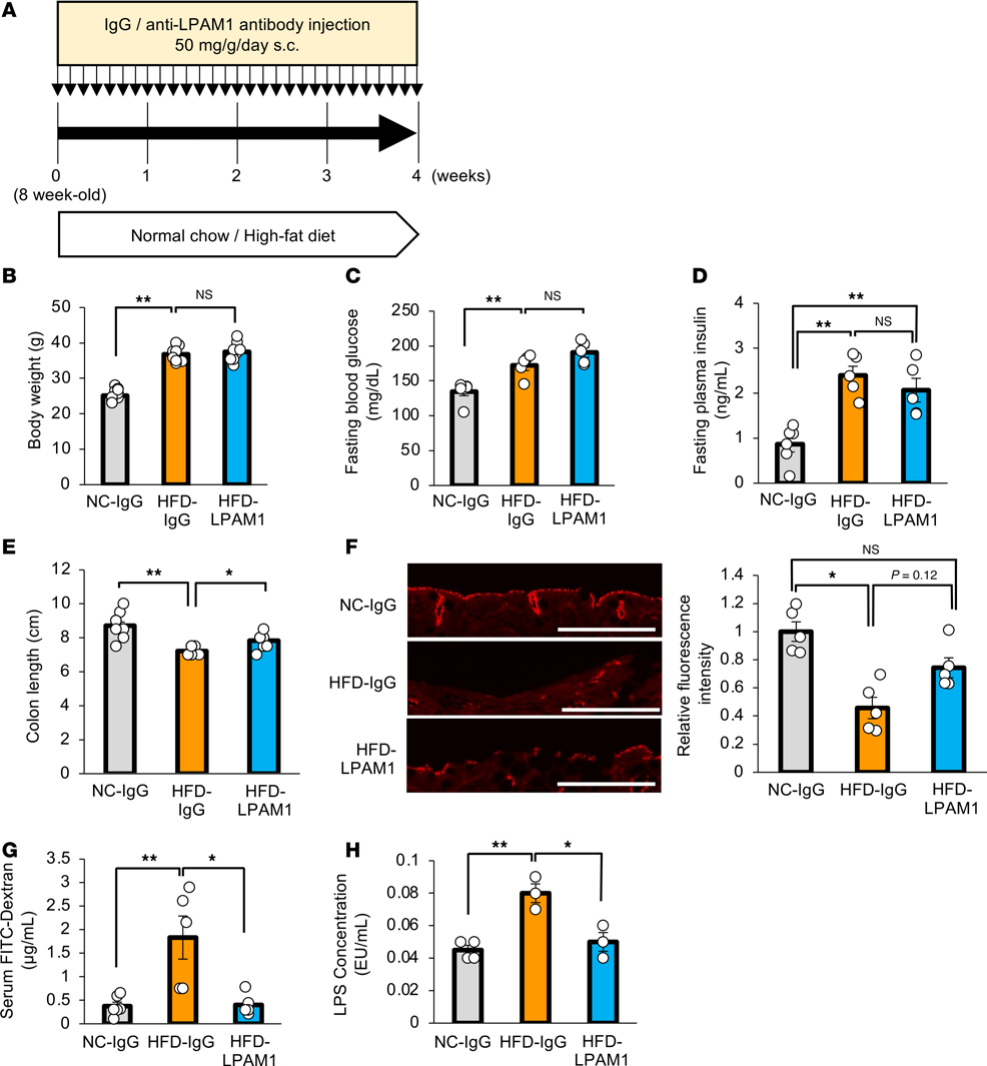
Administration of anti-LPAM1 antibody suppresses colonic inflammation in mice with HFD-induced obesity. (**A**) Scheme of the experimental plan for NC or HFD feeding and antibody administration during the last 2 weeks of NC or HFD feeding. (**B**) Body weight at 4 weeks after antibody-treatment in 8-week-old mice fed NC or HFD (*n* = 11/9/9 per group). (**C**) Fasting blood glucose levels of mice in each group (*n* = 6/5/5 per group). (**D**) Fasting insulin levels of mice are shown (*n* = 6/5/5 per group). (**E**) Colonic lengths of each experimental group (*n* = 11/9/9 per group). (**F**) Photomicrograph image of the immunofluorescence stain in the distal colon and the relative fluorescence intensities in each experimental group (*n* = 5/5/5 per group). Scale bars indicate 100 μm. (**G**) Serum FITC-Dextran levels (4 kDa) after administration to mice in each experimental group (*n* = 6/5/5 per group). (**H**) Serum LPS concentration in the portal vein of mice in each of the experimental groups (*n* = 4/3/3 per group). Data are presented as means ± SEM. *P < 0.05, **P < 0.01 as assessed by 1-way ANOVA, followed by Tukey’s HSD post hoc test.

Previous reports showed the liver-to-pancreas interorgan neuronal relay system to play an important role in β cell proliferation under conditions of obesity (5, 11). Based on these results, we next attempted to block colonic inflammation in HFD-mice and analyzed the impacts of altering factors comprising the neuronal relay system, employing anti-LPAM1 antibody. Anti-LPAM1 antibody or control IgG was continually administered daily to mice in parallel with HFD loading for 4 weeks (Figure 11A). Body weights (Figure 11B) and fasting blood glucose (Figure 11C) and plasma insulin (Figure 11D) levels of HFD-mice treated with either anti-LPAM1 antibody (HFD-LPAM1-mice) or control IgG (HFD-IgG-mice) were similar. On the other hand, colonic shortening was slightly but significantly reversed by anti-LPAM1 antibody administration (Figure 11E). Notably, in HFD-mice, anti-LPAM1 antibody administration suppressed the reduction of colonic occludin expression (Figure 11F) and significantly decreased concentrations of orally administered FITC-Dextran and LPS in the portal vein (Figure 11, G and H). These results indicate that LPAM1 antibody treatment inhibited intestinal barrier disruption in the colons of HFD-mice.

**Figure 12:**
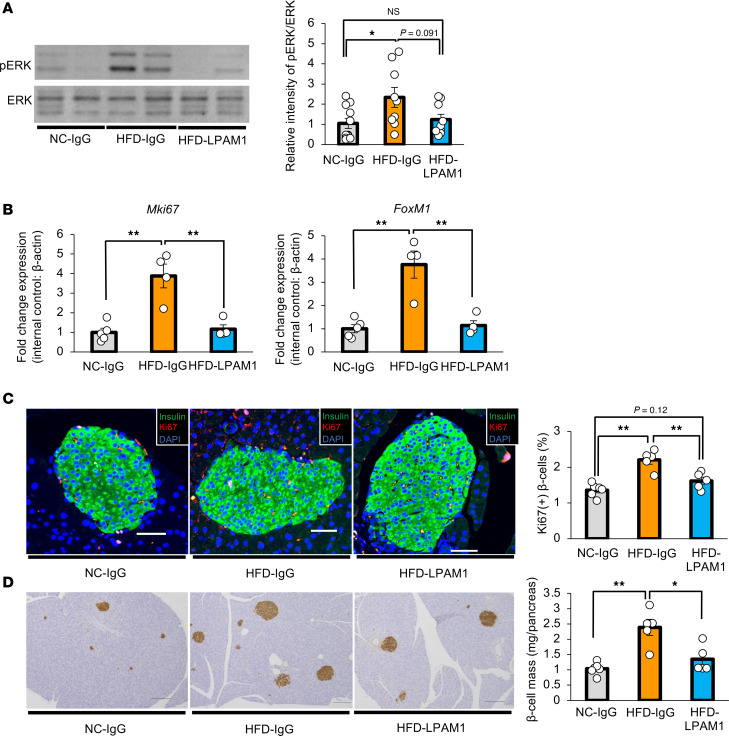
Blockade of colonic inflammation suppresses hepatic ERK activation and β cell proliferation in mice with HFD-induced obesity. (**A**) Representative images and calculated relative intensities of liver extract immunoblotting with anti-ERK and pERK in each group (*n* = 11/9/9 per group). Intensities of bands were calculated as pERK intensity per ERK intensity. (**B**) Expression levels of *FoxM1* and *Mki67* genes in islets isolated from mice in each group (*n* = 5/4/4 per group). (**C**) Representative images and counted Ki67-positive β cell percentage in each of the groups (*n* = 5/5/5 per group). Scale bars indicate 50 μm. (**D**) Representative images and measured β cell masses for each group (*n* = 6/5/5 per group). Scale bars indicate 200 μm. Data are presented as means ± SEM. *P < 0.05, **P < 0.01 as assessed by 1-way ANOVA, followed by Tukey’s HSD post hoc test.

We then examined phosphorylation of hepatic ERK after 4-week anti-LPAM1 antibody administration concurrently with HFD feeding. While hepatic ERK phosphorylation was enhanced in HFD-IgG-mice, treatment with anti-LPAM1 antibody suppressed hepatic ERK phosphorylation in HFD-mice (Figure 12A). In addition, increases in expression of *Mki67* and *FoxM1* were significantly blunted in pancreatic islets isolated from HFD-LPAM1-mice as compared with HFD-IgG-mice (Figure 12B). Consistent with these results, increases in Ki67-positive β cells by HFD loading were suppressed by anti-LPAM1 antibody administration (Figure 12C). Consequently, β cell mass expansion in response to HFD loading was significantly blocked by administering anti-LPAM1 antibody (Figure 12D).

**Figure 13:**
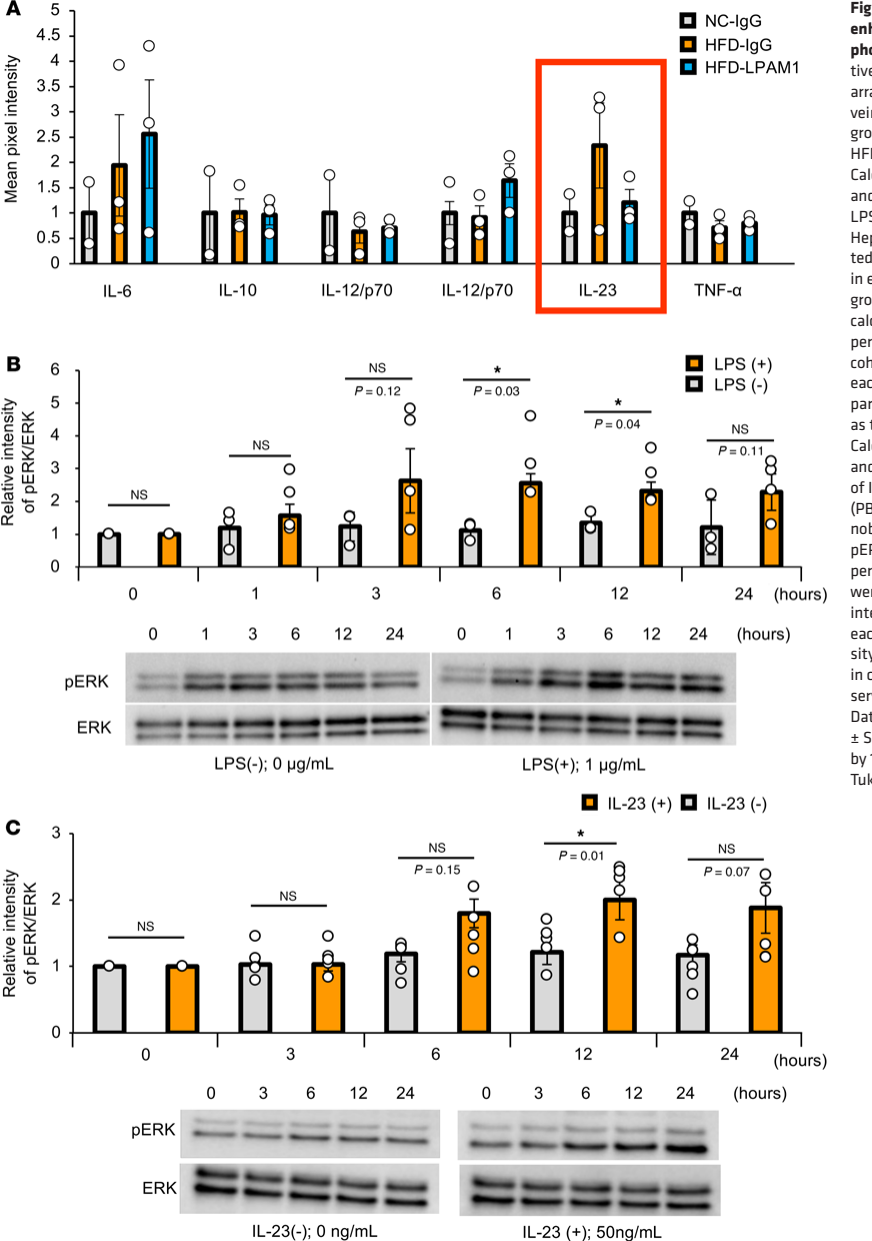
LPS and IL-23 enhances hepatic ERK phosphorylation. (**A**) Representative pixel intensity in cytokine array analyses from the portal vein blood (*n* = 2/3/3 per group in NC-IgG, HFD-IgG, and HFD-LPAM1, respectively). (**B**) Calculated relative intensities and representative images of LPS- or control-treated (PBS) Hepa1-6 extract immunoblotted with anti-ERK and pERK in each group (*n* = 4/3 per group). Band intensities were calculated as pERK intensity per ERK intensity. In each cohort, the band intensity at each hour is shown in comparison with 0 hour, serving as the basal intensity. (**C**) Calculated relative intensities and representative images of IL-23– or control-treated (PBS) Hepa1-6 extract immunoblotted with anti-ERK and pERK in each group (*n* = 5/5 per group). Band intensities were calculated as pERK intensity per ERK intensity. In each cohort, the band intensity at each hour is shown in comparison with 0 hour, serving as the basal intensity. Data are presented as means ± SEM. *P < 0.05 as assessed by 1-way ANOVA, followed by Tukey’s HSD post hoc test.

Finally, we investigated the molecular mechanism(s) by which colonic inflammation leads to hepatic ERK activation. To explore the molecules potentially activating the hepatic ERK pathway under HFD-fed conditions, we performed cytokine array experiments using blood samples obtained from portal veins of NC-IgG-, HFD-IgG-, and HFD-LPAM1- mice. Focusing on cytokines reported to be involved in GI tract inflammation (28), we found interleukin-23 (IL-23) to be increased in the portal veins under HFD conditions but decreased by LPAM1 antibody administration (Figure 13A and Supplemental Figure 4). We therefore examined the effects of IL-23, as well as LPS, which was also increased in the portal veins of HFD-IgG-mice (Figure 11H), on ERK phosphorylation in Hepa1-6 cells, a murine hepatocyte cell line. Both LPS and IL-23 significantly enhanced ERK phosphorylation in Hepa1-6 cells (Figure 13, B and C). In addition, gene expression analyses showed Toll-like receptor-4 (*TLR4*), a receptor for LPS (29), and IL-23 receptor (*mIL23R*) to be abundantly expressed in the liver as compared with other murine tissues, such as the gastrocnemius muscle, pancreatic islets, renal cortex, and brown adipose tissues (Supplemental Figure 5, A and B), suggesting LPS and IL-23 enhance ERK phosphorylation in hepatocytes via these receptors. These results indicate that these proinflammatory factors at least contribute to the activation of the hepatic ERK pathway induced by colonic inflammation.

**Figure 14:**
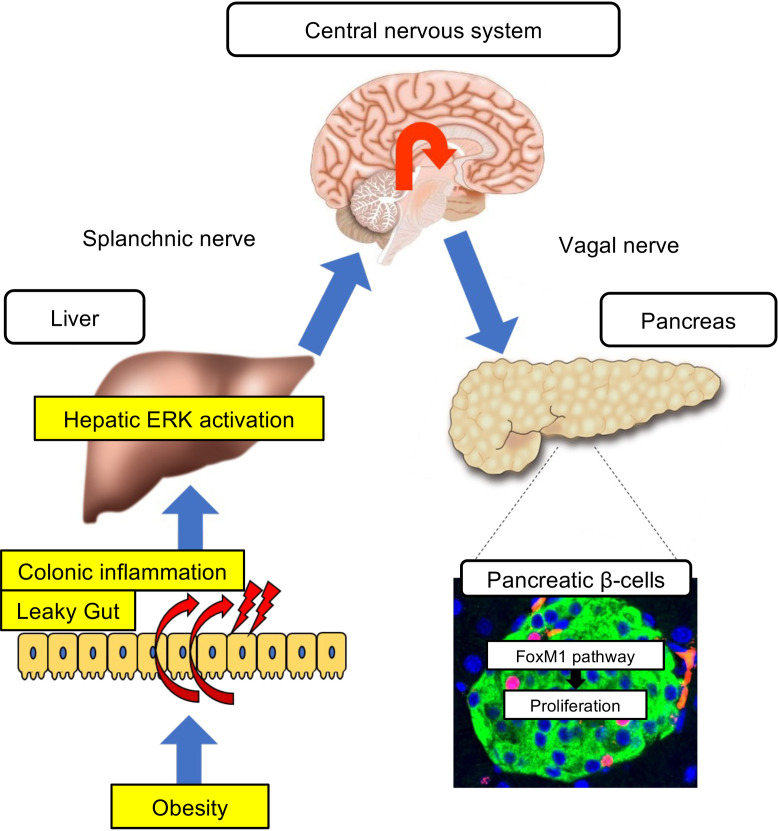
Schematic model. The neuronal relay system from the intestine to the pancreas identified in the present study is depicted graphically.

Taken together, in obesity settings, intestinal barrier disruption in the colon and the associated increases in proinflammatory factors in the portal vein serve as the first trigger for hepatic ERK pathway activation, thereby promoting β cell proliferation (Figure 14).

## Discussion

We previously reported that hepatic ERK activation stimulates the neuronal relay system, consisting of afferent splanchnic nerves from the liver and efferent vagal nerves to the pancreas, leading to adaptive β cell proliferation during obesity development (Figure 1A). Herein, we hypothesized that colonic inflammation under obesity conditions is linked to pancreatic β cell proliferation via this liver-to-pancreas interorgan mechanism. First, to induce colonic inflammation independent of obesity development, we administered DSS. DSS administration induced colonic inflammation accompanied by intestinal barrier disruption in the colon, i.e., produced the so-called leaky gut. Under these conditions, the hepatic ERK pathway was activated, and interestingly, pancreatic β cells showed proliferation with intracellular FoxM1 upregulation, thereby increasing β cell mass. In addition, blockade of hepatic ERK activation, pharmacological inhibition of afferent splanchnic nerves, or surgical dissection of the vagal nerve each inhibited β cell proliferation, indicating β cell proliferation triggered by DSS treatment to be transmitted by a liver-to-pancreas interorgan mechanism similar to that leading to adaptive β cell proliferation during obesity development. In addition, HFD feeding also disrupted the colonic intestinal barrier. Importantly, blockade of leaky gut formation by treatment with anti-LPAM1 antibody suppressed activation of the hepatic ERK pathway and β cell proliferation in both DSS- and HFD-mice. Collectively, these findings indicate that colonic inflammation accompanied by leaky gut formation is both sufficient and necessary for promoting adaptive β cell proliferation (Figure 14).

The GI tract, while being responsible for the absorption of nutrients and fecal excretion, is also an important organ acting as an external barrier, thereby protecting the entire body (14, 15). The GI tract is also considered an important immune organ, where a variety of immune cells function (24) and various inflammatory cytokines are secreted. Indeed, intestinal barrier disruption by HFD loading reportedly allows pro-inflammatory factors to enter the portal vein, which triggers the development of systemic inflammation, leading to insulin resistance (13, 15). As the GI tract is the first systemic site to sense orally ingested foods, the GI tract is suitable for predicting subsequent unfavorable changes possibly elicited by dietary elements, such as HFD. Therefore, it seems reasonable that the gut serves as the first trigger for adaptive β cell proliferation, which may function to prevent hyperglycemia induced by the development of insulin resistance. Bacterial flora in the gut are altered in obesity settings, and conversely, obesity and insulin resistance are also induced by changes in gut microbiota (30). In addition, HFD also alters the immune environment in the GI tract (31). Altered interactions between gut bacteria and the host immune system during obesity development may influence the mechanism, identified in this study, promoting adaptive β cell proliferation.

The liver receives several signals from portions of the GI tract, including the colon, via the portal vein (13, 15, 18). Under conditions of GI tract inflammation, intestinal barrier disruption leads to elevation of a variety of inflammatory factors in the portal vein (18, 32). Indeed, we found that LPS was increased in the portal veins of DSS- and HFD-mice, and these increments were significantly suppressed by anti-LPAM1 antibody treatments (Figure 2F, Figure 7H, Figure 9F, and Figure 11H). In addition, IL-23 levels in the portal veins of HFD-mice showed a trend similar to that observed with LPS (Figure 13A). Furthermore, consistent with a previous report (18), LPS enhanced ERK phosphorylation in Hepa1-6 cells (Figure 13B), and we newly showed that IL-23 also enhanced ERK phosphorylation in these cells (Figure 13C). IL-23 is a cytokine known to modulate the immune system in mucosal tissues, such as the intestine (33). Increased circulating IL-23 concentrations are reportedly associated with metabolic dysfunction–associated steatotic liver disease (34) and LPS-induced acute liver injury (35), Therefore, these inflammatory factors contribute in a coordinated manner to activating the hepatic ERK pathway, although the involvement of additional inflammatory factors remains to be determined.

The present results showed that the interorgan system functions not only in the obese state, which is usually associated with nutritional excess, but also under conditions of colonic inflammation without obesity, which can result in nutritional deficits. Intriguingly, hyperinsulinemia in patients with inflammatory bowel diseases was previously reported (36). This might be a protective mechanism because enhanced insulin signals were reported to support T cell nutrient status, thereby driving optimal T cell effector function acting against increased inflammation (37). Therefore, the leaky gut–triggered neuronal relay may serve as a robust mechanism not only preventing the development of diabetes associated with obesity but also promoting several protective conditions, which manage increased inflammation. Interestingly, insulin levels were reported to be upregulated in patients with Crohn’s disease (38) and ulcerative colitis (39). Therefore, the mechanism identified in the present study may function in patients with inflammatory bowel diseases. Further studies are warranted to elucidate this potential role of the interorgan system.

We have reported several examples of interorgan communication originating in the liver, which maintain metabolic homeostasis and whole-body survival (5, 40–43). As proposed in a previous review (44), the findings of these studies show that the liver perceives the metabolic status of nutrients and transmits information regarding this status to the CNS via neural signals and humoral factors. The liver is the site of direct entry of food absorbed from the intestinal tract, intestinal bacteria-associated cytokines, and hormones from the pancreas, through the portal vein. Therefore, it is reasonable that the liver constantly monitors the immune and inflammatory status of the intestinal tract, as shown in this study, and conveys periphery-derived information. The present study results further support this concept and have shown that information on colonic inflammation is transmitted to the liver via the portal vein, thereby stimulating the interorgan system linking the liver to the pancreas. A similar mechanism whereby the GI microenvironment is sensed by the liver and the sensory inputs are then relayed via the liver-brain-gut system, thereby controlling the number of regulatory T cells in the gut and maintaining gut homeostasis, was recently reported (24). Thus, the liver may monitor gut conditions and send afferent signals to maintain allostasis of the immune system as well as glucose metabolism.

In conclusion, we identified leaky gut as the first trigger of β cell proliferation during obesity development. This mechanism is governed by an interorgan network involving several tissues/organs, including the colon, the liver, the CNS, and pancreatic islets. The present study revealed a potentially novel role of the GI tract in maintenance of glucose homeostasis via regulation of β cell mass.

## Methods

### Sex as a biological variable.

Our study examined male mice because male animals exhibited less variability in phenotype. It is unknown whether the findings are relevant for female mice.

### Animals.

C57BL/6N male mice purchased from SLC Japan were used for this research. At 8 weeks of age, each mouse underwent the following interventions. Mice were fed NC (4.9% fat; MF, Oriental Yeast Co. Ltd.) or HFD (60% fat; D12492, Open Source Diets, Research Diets). All mice were housed in a controlled environment (room temperature 25°C) with a 12-hour light/12-hour dark cycle.

### Operative interventions of animals or sacrificing.

For the operative interventions and animal sacrifice procedure, anesthesia was administered with an intraperitoneal injection containing a mixture of medetomidine (0.3 mg/kg), midazolam (4 mg/kg), and butorphanol tartrate (5 mg/kg).

### DSS treatment.

To create a leaky gut model, 1.5% DSS (19, 45) (MP Biomedicals) diluted in the drinking water was provided. For a control group, the same water without DSS was provided.

### Assessment of colonic inflammation and intestinal permeability.

The severity of colonic inflammation was evaluated by measuring colonic shrinkage and using the disease activity score, based on a previous report (19). Additionally, histological evaluations were performed according to a previous report, by measuring the epithelial fluorescence intensity of occludin protein, the major tight junction protein, on the colon (46) using BIOREVO BZ-X710 and BZ-II Analyzers (Keyence).

DSS-induced colonic inflammation tends to involve the distal portion of the colon (19). Therefore, the colonic sample was equally divided into proximal and distal portions, and we evaluated the distal specimen. The same procedure was performed when assessing HFD-treated mice. Precise pathological procedures are described in the *Histological analysis and immunohistochemistry* section.

To evaluate real-time intestinal permeability, HFD-treated mice (47) and DSS-mice (48) were orally provided 4,000 or 40,000 Da FITC-Dextran (Chondrex), respectively, as previously reported, via a nasogastric tube at 20 mL/kg after being fasted for 4 hours. The impacts on permeability in each mouse model were then assessed. Blood samples were collected from the portal vein after 3 hours. The concentration of FITC from obtained serum was measured by fluorescence spectrophotometry (excitation at 490 nm and emission at 520 nm) using a GloMax Discover Microplate Reader (Promega). These protocols were carried out according to the official protocol provided by Chondrex.

### β cell mass measurement.

Excised whole pancreatic tissues were fixed with 10% formalin at 4°C overnight and embedded in paraffin. Pancreatic sections, 3 μm in thickness, were made at an interval of 100 μm, then immunostained with insulin antibody (I2018, MilliporeSigma). Five sections per sample were analyzed by randomly selecting and observing sections of the entire pancreas from head to tail as described previously (49). Immunoreactivity was then visualized by incubation with a substrate solution containing 3,3′diaminobenzidine tetra-hydrochloride. After immunostaining, total pancreatic and insulin-positive areas in each section were measured using BIOREVO BZ-X710 (version 1.4.0.1) and BZ-X700 Analyzers (version 1.4.1.1) (Keyence). We then determined the β cell mass, calculating the average ratio of the total insulin-positive area to the total pancreatic area, and multiplied this ratio by total pancreatic weight.

### Histological analysis and immunohistochemistry.

Pancreatic or intestinal tissues were excised and fixed in 10% formalin at 4°C overnight and thereafter embedded in paraffin. Pancreatic sections, 3 μm in thickness, were made at an interval of 100 μm. These samples were similarly excised and fixed in 10% formalin at 4°C overnight and thereafter embedded in paraffin as sections 3 μm in thickness made at an interval of 20 μm. Some sections were initially stained with hematoxylin and eosin. Collected intestinal tissues were immediately washed with cold phosphate-buffered saline to eliminate stool and stored as Swiss-rolled specimens (50).

For immunostaining, specimens were stained according to the goal of each analysis with the appropriate primary antibodies: β cell mass insulin (IR002, Dako), BrdU in situ detection (Kit 551321, BD Biosciences), Ki67 in situ detection (ab15580, Abcam), and occludin immunointensity measurement (OC-3F10, 33-1500, Invitrogen). Alexa Fluor 647 donkey anti-guinea pig IgG (706-605-148, Jackson ImmunoResearch), Alexa Fluor 488 donkey anti-mouse IgG (715-545-151, Jackson ImmunoResearch), and Alexa Fluor 594 donkey anti-rat IgG (712-585-153, Jackson ImmunoResearch) were used as secondary antibodies. DAPI (D9542, MilliporeSigma) was used for nuclear staining. For β cell analysis, at least 20 islets and over 1,000 nuclei per sample were counted. To measure cell proliferation, 0.8 mg/mL BrdU (Carbosynth) diluted in drinking water was administered for 1 week during the interventions. In accordance with prior reports (51, 52), to measure epithelial occludin intensity on the distal colon, 3 randomly chosen points were measured and averaged per mouse. Control mice were also analyzed using the corresponding sections. Counting processes were performed with BIOREVO BZ-X710 and BZ-X700 Analyzers (version 1.4.1.1) (Keyence).

### LPS measurement in the portal vein.

A fine catheter was used to puncture the portal vein, and blood samples were carefully collected without hemolysis. Sampling was conducted after 7 hours of fasting, before insertion. Serum samples were mixed separately with Limulus amoebocyte lysate reagent and then incubated at 37°C for 30 minutes, after which the activities were determined with a quantitative chromogenic assay. Plasma LPS concentration was quantitatively measured with Endotoxin-specific Endospecy kit (Seikagaku Kogyo).

### Immunoblotting.

Liver samples were homogenized in lysis buffer containing 100 mM Tris at pH 8.5, 250 mM NaCl, 1 mM EDTA, 10% glycerol, 1% Nonidet P-40, 1 mM sodium orthovanadate, 2 mM phenylmethylsulfonyl fluoride, 40 mM β-glycerophosphate, 50 mM NaF, 2 μg/mL aprotinin, and 2 μg/mL leupeptin using MicroSmash MS-100 for 30 seconds at 4,000 rpm (TOMY). As for Hepa1-6 cell lines (ATCC), protein samples were collected as previously reported (53). Tissue homogenates or cell-derived samples were centrifuged for 10 minutes at 14,000*g*, and the supernatants including tissue protein extracts were boiled in Laemmli buffer containing 10 mM dithiothreitol, then subjected to SDS-PAGE. Separated proteins were transferred to nitrocellulose membranes and blocked in Tris-buffered saline containing 3% fetal bovine serum. Immunoblot analyses were performed using antibodies against ERK (9102, Cell Signaling Technology), p-ERK (4376, Cell Signaling Technology), and actin (A2066, MilliporeSigma) at 1:5,000, 1:2,500, and 1:5,000, respectively. Anti-rabbit IgG HRP-linked donkey F(ab)2 fragment (NA9310 and NA9340, GE Healthcare, now Cytiva) was used as the secondary antibody. Band visualization was performed with Pierce ECL Plus Western Blotting Substrate (Thermo Fisher Scientific), and quantitative data were obtained employing a ChemiDoc Touch Imaging System (Bio-Rad Laboratories). Band intensities were calculated using the ImageJ program (National Institutes of Health). Immunoblotting of the liver samples was performed as previously reported (54).

### Blood glucose and insulin measurements.

Blood glucose levels were measured with Glutestmint (Sanwa Kagaku Kenkyusho). Before the measurements of fasting blood glucose levels, mice were fasted for more than 7 hours as previously reported (53). Plasma insulin levels were measured using a mouse/rat insulin ELISA kit (Morinaga) as described previously (55).

### Islet isolation study.

Islets were isolated from control and DSS- or HFD-mice at the completion of the interventions by retrograde injection of cold Hanks’ balanced salt solution containing 1.0 mg/mL collagenase V (MilliporeSigma) into the pancreatic duct. The pancreases were digested for 8.5 minutes at 37°C, and for collection, the islets were hand-picked under a microscope (SMZ800, Nikon) as described previously (56).

### Quantitative real-time PCR analysis.

Tissue samples were homogenized with MicroSmash MS-100 for 30 seconds at 4,000 rpm (TOMY). Total RNA was extracted from isolated mouse islets, liver, gastronomic muscle, brown adipose tissue, and renal cortex with an RNeasy Micro Kit (QIAGEN). Employing a 100 ng quantity of RNA, cDNA synthesis was performed with a ReverTra Ace qPCR RT Master Mix (Toyobo). Real-time PCR was performed using the CFX96 Touch Real-Time PCR Detection System (Bio-Rad Laboratories) as described previously (43, 57).

The relative amount of mRNA was calculated with *Actb* as the invariant control using the Ct method. Primer sequences were as follows: *Actb*, forward, 5′-GATGCCCTGAGGCTCTT-3′; *Actb*, reverse, 5′-TGTGTTGGCATAGAGGTCTTTAC-3′; *FoxM1*, forward, 5′-GCTCCATAGAAATGTGACCATC-3′; *FoxM1*, reverse, 5′-AACCTTCACTGAGGGCTGTAAC-3′; *Mki67*, forward, 5′-ATCCAGATGTTAGGCGTTTGAG-3′; *Mki67*, reverse, 5′-ACAGTTTCACTTTTCTGGTGACTTC-3′; *TLR4*, forward, 5′- TTATCCAGGTGTGAAATTGAAACAATTG-3′; *TLR4*, reverse. 5′-CACAGCCACCAGATTCTCTAAACTT-3′; *mIL23R*, forward, 5′- GCTCGGATTTGGTATAAAGG-3′ and *mIL23R*, reverse, 5′- ACTTGGTATCTATGTAGGTAGG-3′

### In vivo antibody administrations.

Mice were given subcutaneous injections of 25 μg/g/d anti-α_4_β_7_ integrin antibody (BE0034, DATK32, Bio X Cell) into the loose skin over the neck as previously reported (32, 58). The DSS-treated mice were also concomitantly administered DSS for 7 days. For HFD mice, 50 μg/g/d of the same anti-α_4_β_7_ integrin antibody were similarly administered during the entire period of HFD feeding (4 weeks). For the control group, the same amount of Rat IgG2A (BE0089, 2A3, Bio X Cell) was similarly administered.

### Recombinant adenovirus.

Recombinant adenoviruses were cultured in HEK293 cells (ATCC) and purified by cesium chloride gradient ultracentrifugation for 2 hours at 35,000 rpm. Viral titers were determined using an endpoint cytopathic effect assay. Thereafter, 5 × 10^8^ PFU per mouse of the adenovirus encoding the dominant-negative mutant of the mouse MEK-1 gene for hepatic ERK suppression (d/nMEK) were intravenously injected. The controls received 1 × 10^8^ PFU per mouse of LacZ adenovirus as previously reported (54).

### Selective blockade of afferent splanchnic nerve.

Seven days before DSS treatment, mice were subjected to selective afferent splanchnic nerve blockade. Splanchnic nerves form a sheath along the celiac artery and contain afferent fibers from the hepatobiliary system. For the selective deafferentation of the spinal nerve from the liver, we applied capsaicin to the splanchnic nerve as previously described (5). A laparotomy incision was made on the ventral midline, and the celiac artery was exposed. The celiac artery was wrapped in gauze immersed with or without capsaicin (MilliporeSigma) dissolved in olive oil (5% wt/vol) for 30 minutes, and thereafter the surgical incision was closed.

### Subdiaphragmatic vagotomy.

A laparotomy incision was made on the ventral midline. For subdiaphragmatic vagotomy, both the ventral and dorsal vagal trunks were separated from the esophagus, and both the ventral vagal trunk below the bifurcation of the hepatic branch and the dorsal vagal trunk were transected as previously reported (5, 9–11). The sham operation was performed using an identical procedure but with the nerves left intact. At 7 days after these operations, interventions, including islet isolation and extraction of the pancreas, were carried out.

### Insulin tolerance tests.

Insulin tolerance tests were performed as previously reported with regular insulin (0.5 U/kg of body weight) (56).

### Cell culture.

Hepa1-6 cells were purchased from ATCC (CRL-1830) and cultured as previously reported (53). After reseeding and overnight culture, LPS at 1.0 mg/mL, recombinant IL-23 at 50 ng/mL, or PBS, as a control, was administered into the cell culture medium. The dissociated cells were then collected after washing with PBS, as previously reported (53).

LPS and recombinant mouse IL-23 were purchased from MilliporeSigma and R&D Systems, Bio-Techne, respectively.

### Cytokine array.

Portal vein blood samples were collected from the mice after overnight fast and immediately centrifuged at 4°C for 10 minutes at 2,000*g*. The supernatants (plasma) were applied to the cytokine array. Cytokine array was performed with a Proteome Profiler Mouse Chemokine Array Kit (R&D Systems, Bio-Techne) in accordance with a previous report (57) and the manufacturer’s instructions.

### Statistics.

All data are expressed as means ± SEM. The statistical significance of differences between 2 groups was assessed by applying the 2-tailed unpaired *t* test. For experiments yielding data requiring multiple comparisons, we applied 1-way ANOVA followed by Tukey’s HSD post hoc test. Differences were regarded as significant at *P* < 0.05. Statistical analysis was performed using BellCurve for Microsoft Excel. Sample size was based on previous studies in the field and pilot experiments. A reasonable sample size was estimated to perform valid statistical analysis and to ensure the reproducibility of the results. Blinding was not required as the same analysis was adopted for both experimental and control groups in all experimental conditions, and as the data analyses were based on objectively measurable data.

### Study approval.

Animal studies were conducted in accordance with the Tohoku University institutional guidelines. Ethics approval was obtained from the Institutional Animal Care and Use Committee of the Tohoku University Environmental & Safety Committee.

### Data availability.

The main data supporting the results in this study are available within the paper, supplement, and Supporting Data Values file.

## Author contributions

H Kubo, JI, and TI conducted the research and obtained the data, contributed to relevant discussions, wrote the manuscript, and reviewed/edited the manuscript. MK, YK, AE, HS, JS, TH, H Komamura, TS, SH, YA, SK, KT, and KK obtained the data and contributed to relevant discussions. H Katagiri contributed to the relevant discussions, writing the manuscript, and reviewing/editing the manuscript.

## Supplementary Material

Supplemental data

Unedited blot and gel images

Supporting data values

## Version 1 05/08/2025

Electronic publication
