# Supplementary material for: Colonic inflammation triggers β cell proliferation during obesity development via a liver-to-pancreas interorgan mechanism
Source: JCI Insight. 2025 May 8;10(9):e183864. doi: 10.1172/jci.insight.183864 (PMC12128978; doi:10.1172/jci.insight.183864)
Supplement: Supplemental data [file jciinsight-10-183864-s058.pdf]

Colonic inflammation triggers β-cell proliferation during obesity development via a liver-to-pancreas inter-organ mechanism

Haremaru Kubo, Junta Imai¶, Tomohito Izumi, Masato Kohata,
Yohei Kawana, Akira Endo, Hiroto Sugawara, Junro Seike, Takahiro Horiuchi,
Hiroshi Komamura, Toshihiro Sato, Shinichiro Hosaka, Yoichiro Asai,
Shinjiro Kodama, Kei Takahashi, Keizo Kaneko and Hideki Katagiri¶

Contents

Supplemental Figure 1.
Supplemental Figure 2.
Supplemental Figure 3.
Supplemental Figure 4.
Supplemental Figure 5.

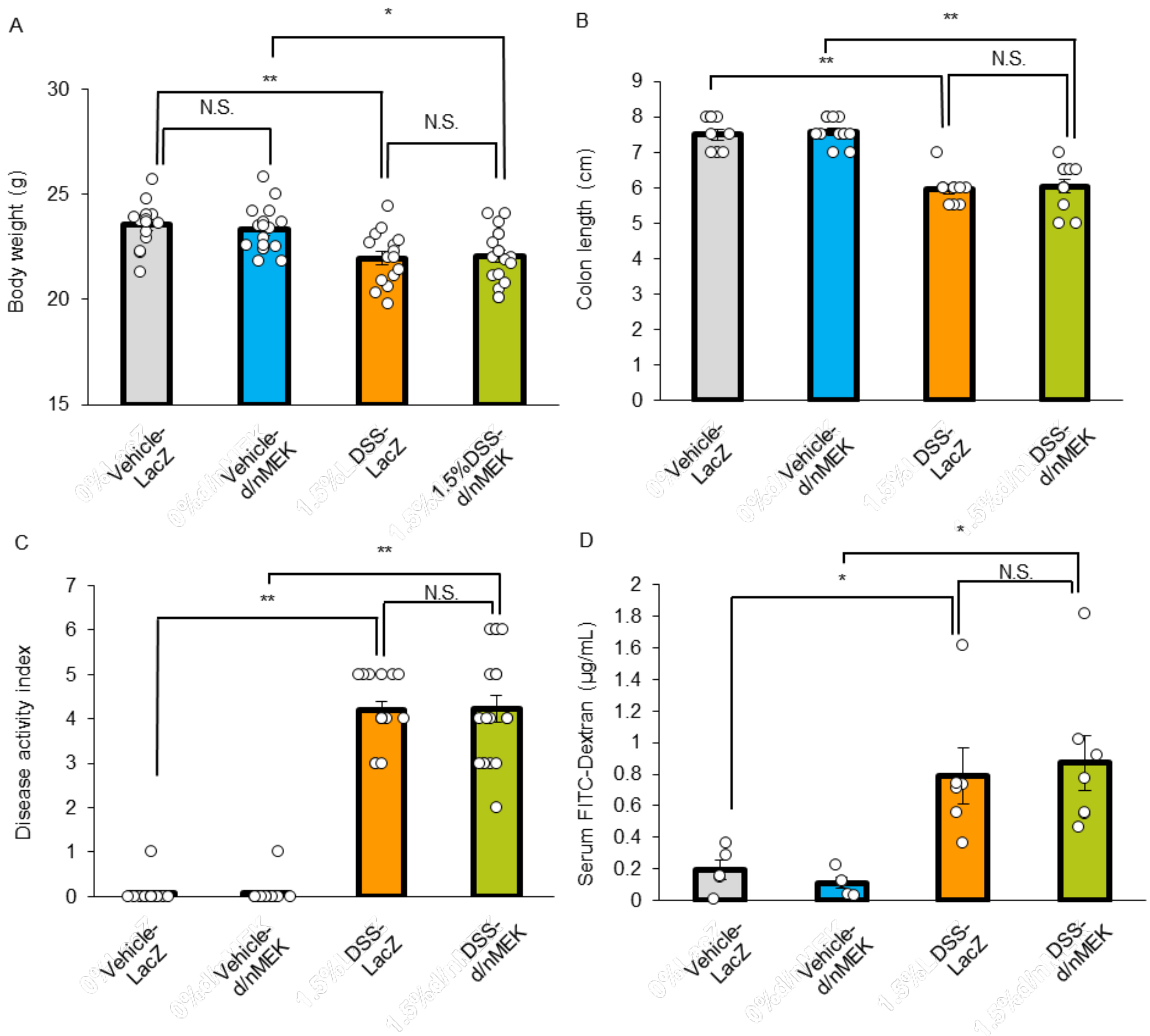

Supplemental Figure 1

Administration of d/nMEK adenovirus neither suppressed colonic inflammation nor intestinal barrier disruption in DSS-mice

(A) Body weights at day 7 of the four experimental groups treated with 0% or 1.5% dextran sulfate sodium (DSS) and adenovirus administration (LacZ or dominant negative MEK(d/nMEK)); 0% DSS-LacZ (Vehicle-LacZ), 0% DSS-d/nMEK (Vehicle-d/nMEK), 1.5%DSS-LacZ (DSS-LacZ) and 1.5%DSS-d/nMEK (DSS-d/nMEK). (n = 15/18/20/23 per group, respectively, and the order is the same hereafter).

(B) Colonic lengths of each of the experimental groups are shown (n = 10/12/10/12 per group).

(C) Disease activity indexes are shown for each experimental group (n = 15/15/15/18 per group)

(D) Serum fluorescein isothiocyanate dextran (FITC)-Dextran levels (40 kDa) after administration to mice in each experimental group are shown (n = 5/5/6/7 per group).

Data are presented as means ± SEM. $^{*}P < 0.05$, $^{**}P < 0.01$ as assessed by one-way ANOVA, followed by Tukey's HSD post hoc test. N.S.; no significant

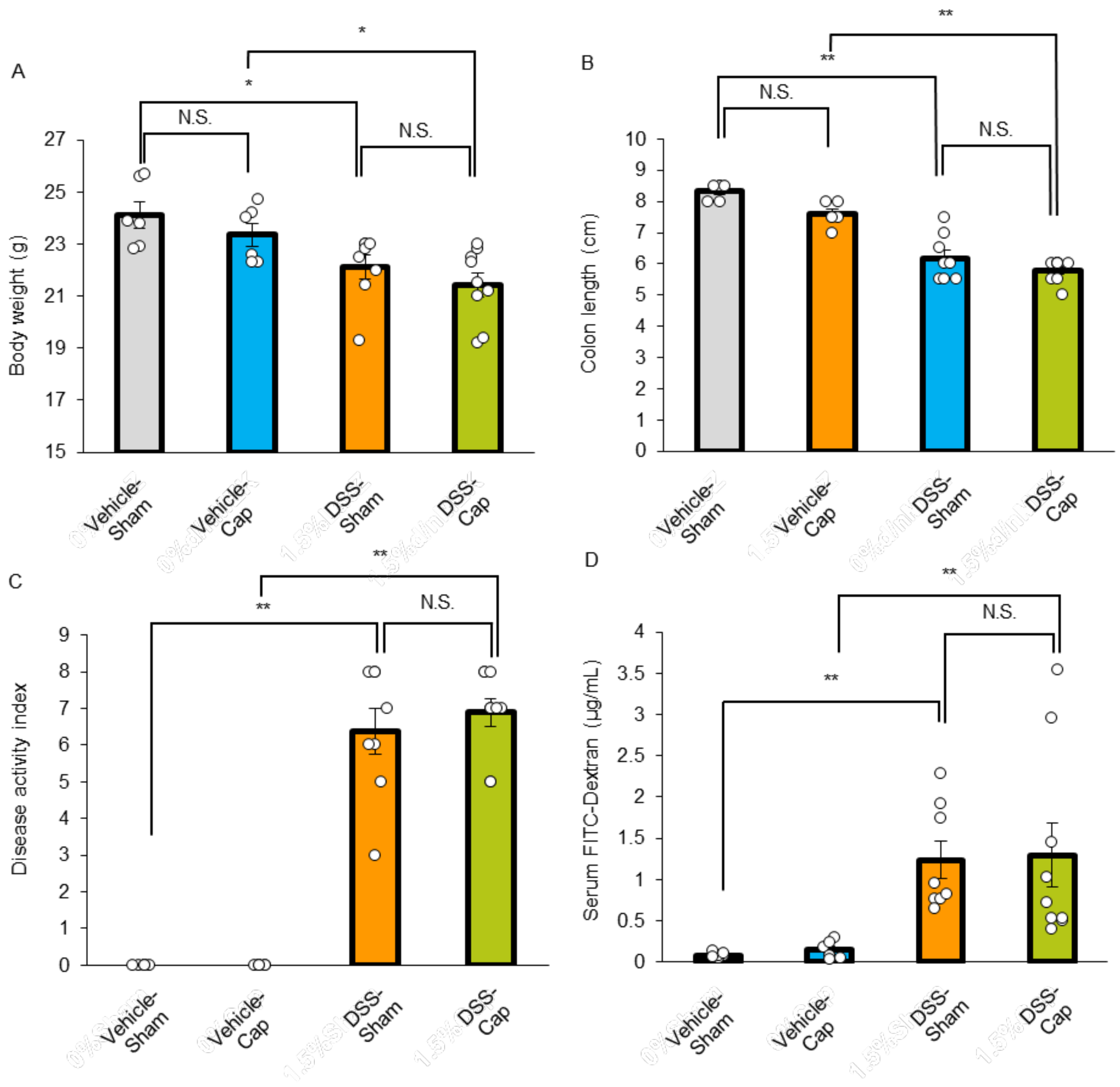

Supplemental Figure 2

Capsaicin treatment neither suppressed colonic inflammation nor intestinal barrier disruption in DSS-mice

(A) Body weights at day 7 in the four experimental groups treated with 0% or 1.5% dextran sulfate sodium (DSS) and sham operation or capsaicin treatment conducted one week before DSS-treatment; 0% DSS-sham operation (Vehicle-Sham), 0% DSS-capsaicin (Vehicle-Cap), 1.5%DSS-sham operation (DSS-Sham) and 1.5%DSS-capsaicin (DSS-Cap) (n = 6/6/8/9 per group, respectively, and the order is the same hereafter).

(B) Colonic lengths of each of the experimental groups are shown (n = 6/6/8/9 per group).

(C) Disease activity indexes are shown for each experimental group (n = 6/6/8/9 per group)

(D) Serum fluorescein isothiocyanate dextran (FITC)-Dextran levels (40 kDa) after administration to mice in each experimental group are shown (n = 6/6/8/9 per group).

Data are presented as means ± SEM. *P < 0.05, **P < 0.01 as assessed by one-way ANOVA, followed by Tukey's HSD post hoc test. N.S.; no significant

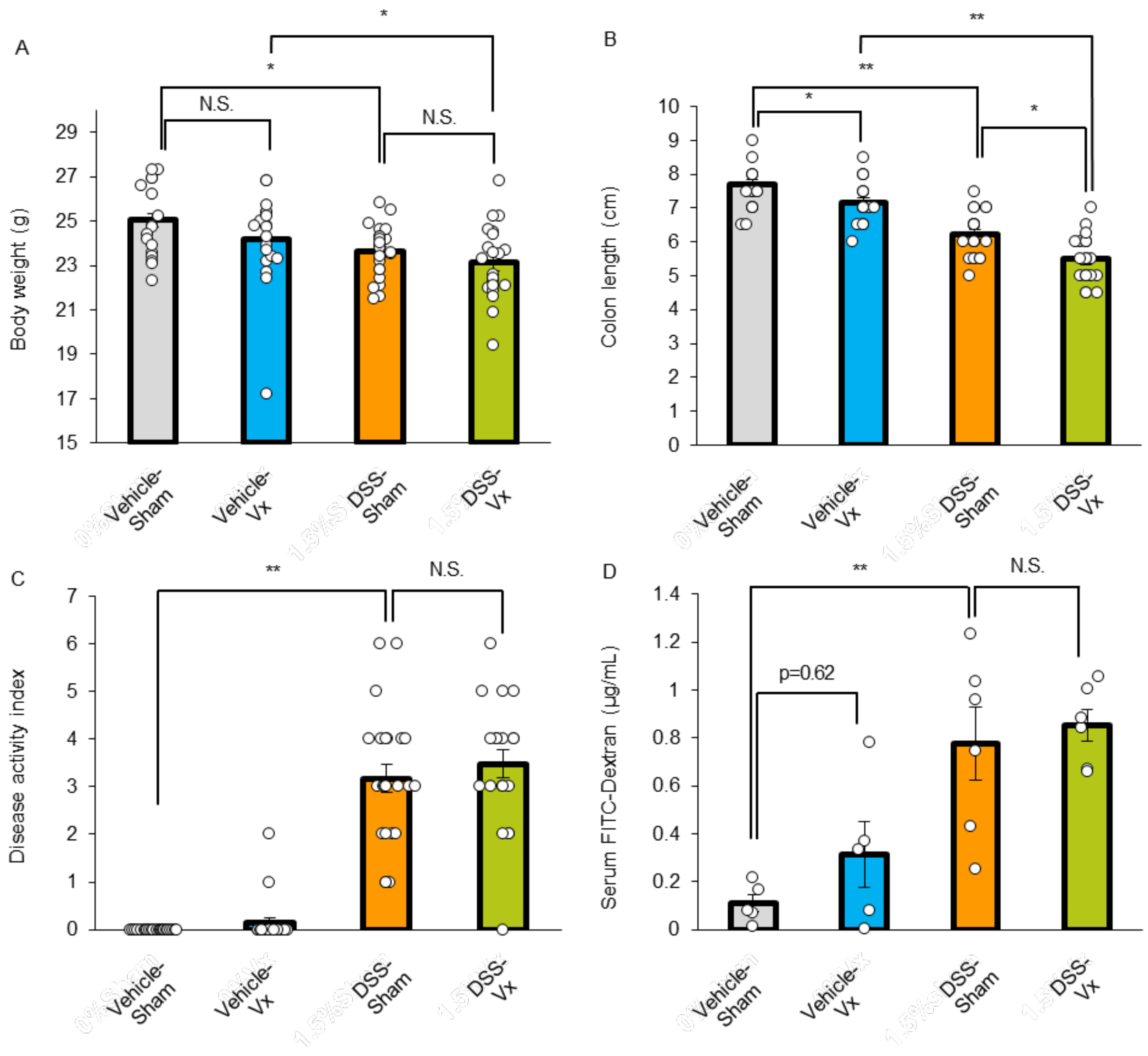

Supplemental Figure 3

Subdiaphragmatic vagotomy neither suppressed colonic inflammation nor intestinal barrier disruption in DSS-mice

(A) Body weights at day 7 in the four experimental groups treated with 0% or 1.5% dextran sulfate sodium (DSS) and sham operation or vagotomy conducted one week before DSS-treatment; 0% DSS-sham operation (Vehicle-Sham), 0% DSS-vagotomy (Vehicle-Vx), 1.5%DSS-sham operation (DSS-Sham) and 1.5%DSS-vagotomy (DSS-Vx) (n = 23/20/23/21 per group, respectively, and the order is the same hereafter).

(B) Colonic lengths of each of the experimental groups are shown (n = 23/20/23/21 per group).

(C) Disease activity indexes are shown for each experimental group (n = 23/20/23/21 per group)

(D) Serum fluorescein isothiocyanate dextran (FITC)-Dextran levels (40 kDa) after administration to mice in each experimental group are shown (n = 5/5/7/7 per group).

Data are presented as means ± SEM. *P < 0.05, **P < 0.01 as assessed by one-way ANOVA, followed by Tukey's HSD post hoc test. N.S.; no significant

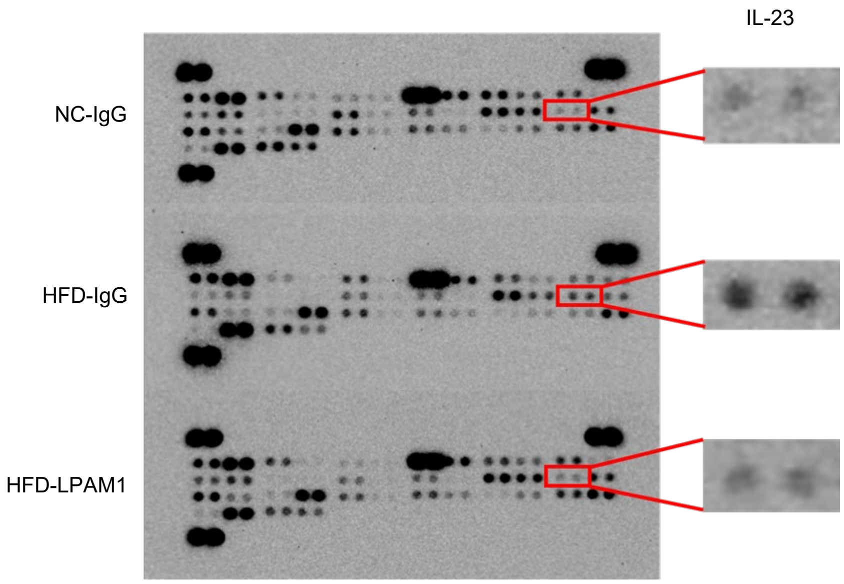

Supplemental Figure 4

Cytokine arrays using portal vein blood samples of NC-IgG-, HFD-IgG- and HFD-LPAM1- mice.

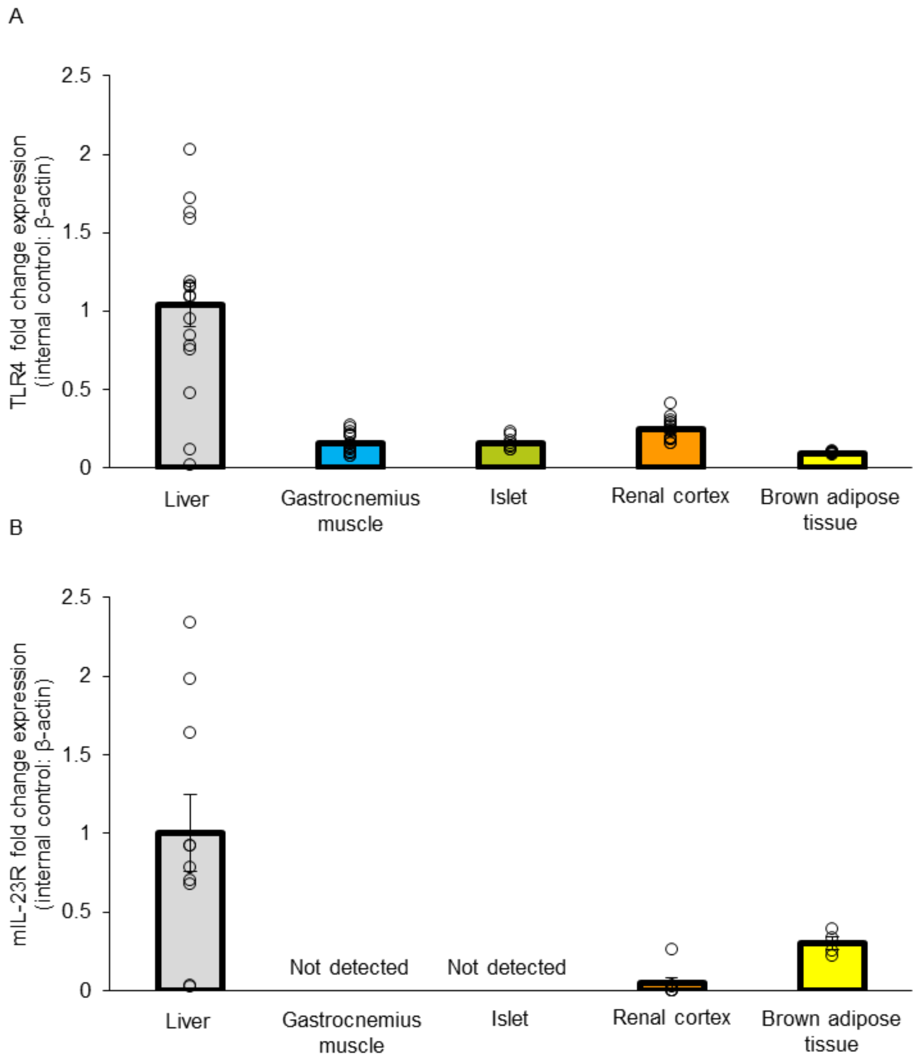

Supplemental Figure 5

The relative expressions of *TLR4* (A) and *mIL-23R* (B) in the liver, the gastrocnemius muscle, pancreatic islets, the renal cortex and brown adipose tissues of mice. A: n=16/16/8/16/8, B; n=10/8/8/7/4, respectively. The data indicate relative expressions (mean expression in the liver, i.e., the control, was defined as 1.0).
