# Supplementary material for: Colonic inflammation triggers β cell proliferation during obesity development via a liver-to-pancreas interorgan mechanism
Source: JCI Insight. 2025 May 8;10(9):e183864. doi: 10.1172/jci.insight.183864 (PMC12128978; doi:10.1172/jci.insight.183864)
Supplement: Unedited blot and gel images [file jciinsight-10-183864-s059.pdf]

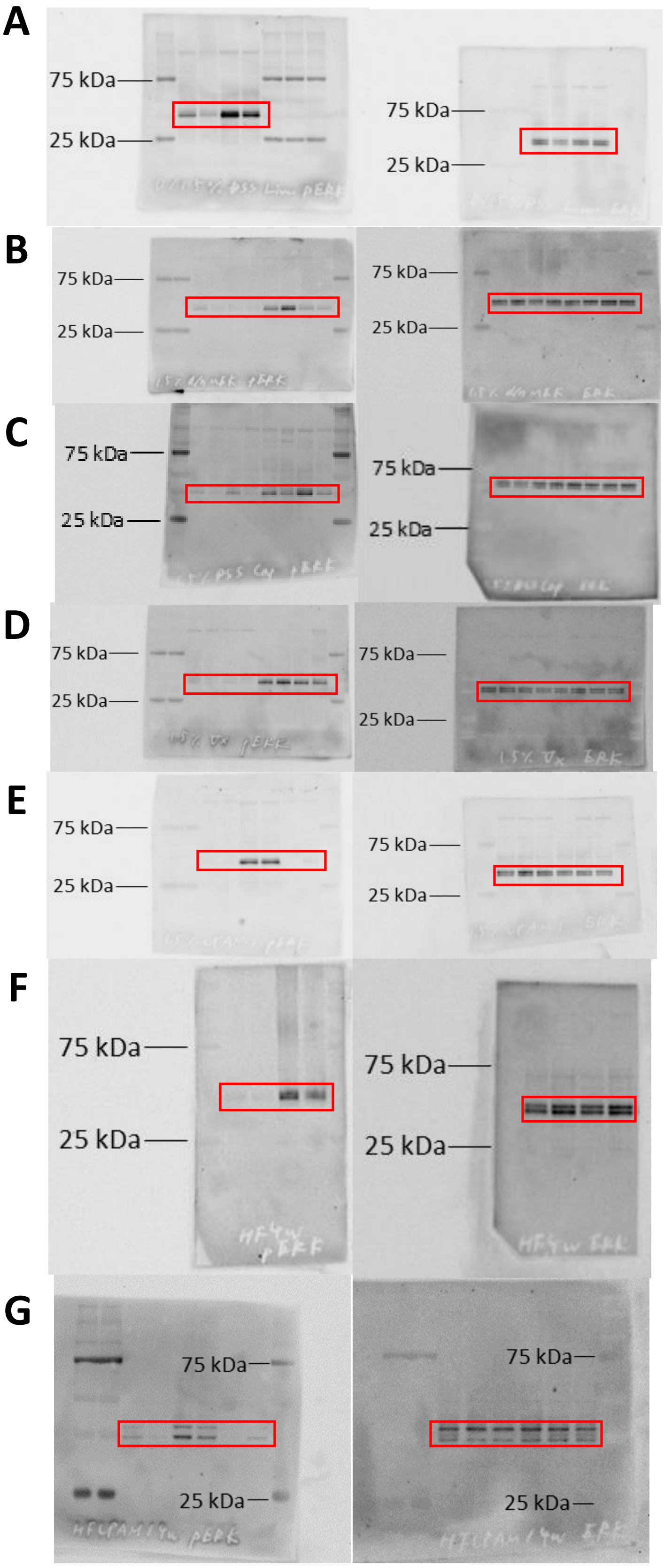
A
75 kDa
25 kDa
75 kDa
25 kDa
B
75 kDa
25 kDa
75 kDa
25 kDa
C
75 kDa
25 kDa
75 kDa
25 kDa
D
75 kDa
25 kDa
75 kDa
25 kDa
E
75 kDa
25 kDa
75 kDa
25 kDa
F
75 kDa
25 kDa
75 kDa
25 kDa
G
75 kDa
25 kDa
75 kDa
25 kDa

(A) Images of the entire immunoblotted-membranes which are inserted in Figure 3A.
(B) Images of the entire immunoblotted-membranes which are inserted in Figure 4B.
(C) Images of the entire immunoblotted-membranes which are inserted in Figure 5C.
(D) Images of the entire immunoblotted-membranes which are inserted in Figure 6C.
(E) Images of the entire immunoblotted-membranes which are inserted in Figure 8A.
(F) Images of the entire immunoblotted-membranes which are inserted in Figure 10A.
(G) Images of the entire immunoblotted-membranes which are inserted in Figure 12A.

(A) To (G) Left: Immunoblotting with anti-phosphorylated ERK antibodies, Right: Immunoblotting with anti-ERK antibodies. In each immunoblotting for the same experiment, the same amounts of samples were loaded onto separate membranes, and electrophoresis was performed simultaneously.

A

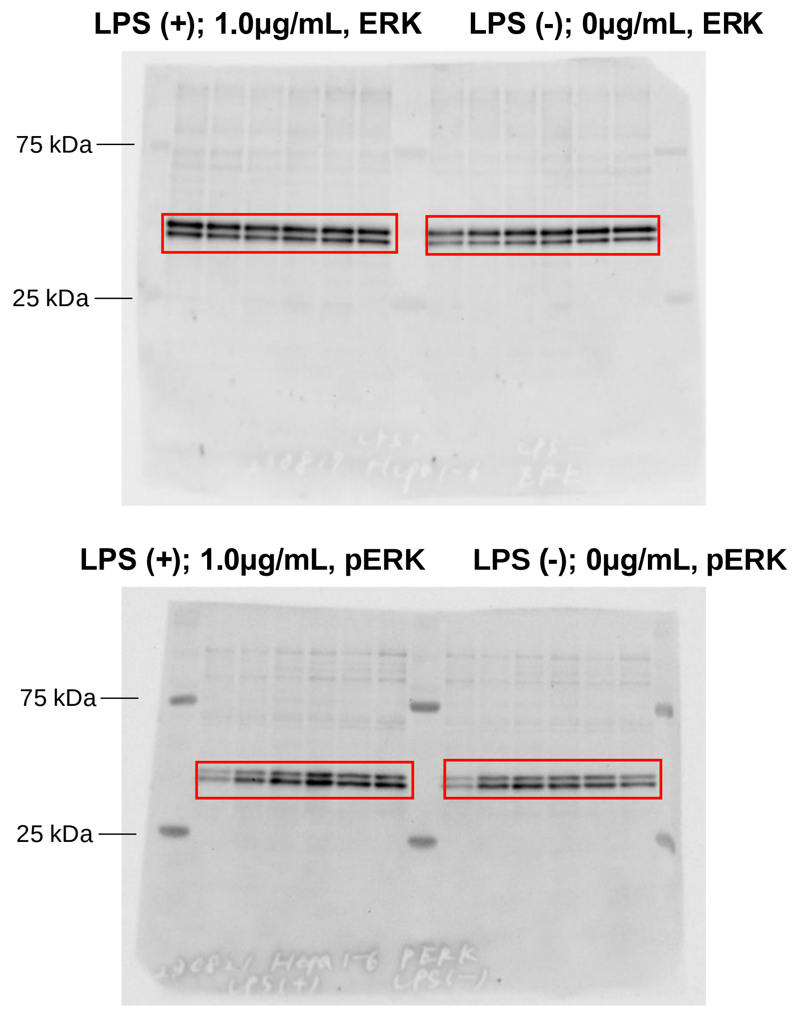
LPS (+); 1.0μg/mL, ERK
LPS (-); 0μg/mL, ERK
75 kDa
25 kDa
LPS (+); 1.0μg/mL, pERK
LPS (-); 0μg/mL, pERK
75 kDa
25 kDa

B

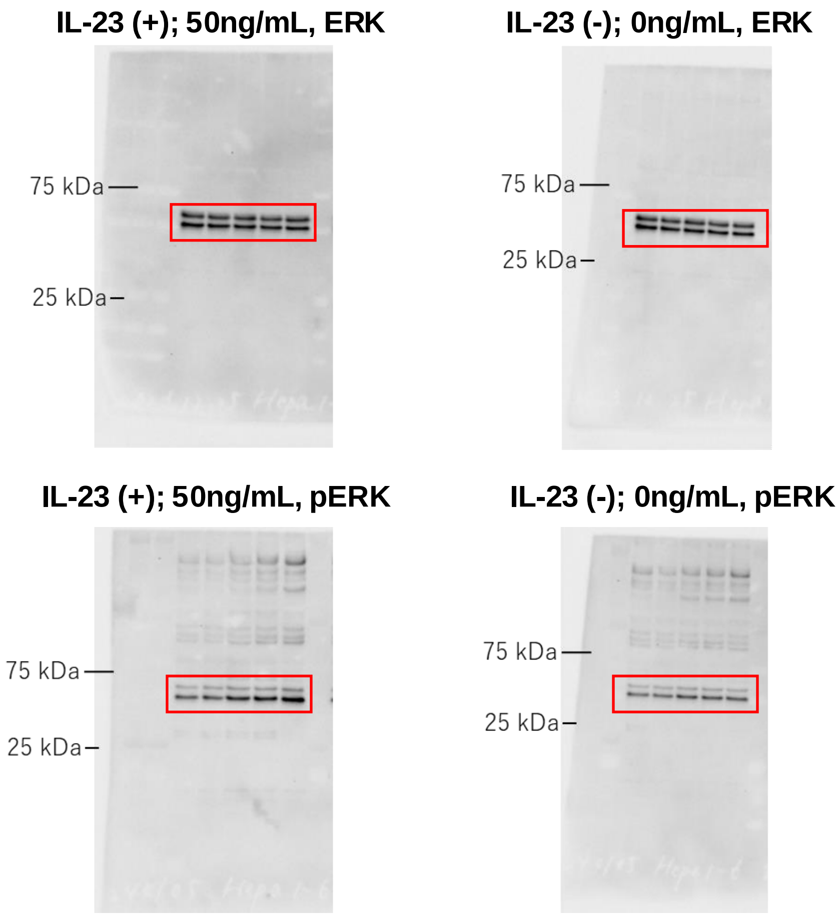
IL-23 (+); 50ng/mL, ERK
IL-23 (-); 0ng/mL, ERK
75 kDa
25 kDa
75 kDa
25 kDa
IL-23 (+); 50ng/mL, pERK
IL-23 (-); 0ng/mL, pERK
75 kDa
25 kDa
75 kDa
25 kDa

(A) Images of the entire immunoblotted-membranes which are inserted in Figure 13B.

(B) Images of the entire immunoblotted-membranes which are inserted in Figure 13C.

In each immunoblotting for the same experiment, the same amounts of samples were loaded onto separate membranes, and electrophoresis was performed simultaneously.
